# Differential Preference of *Burkholderia* and *Mesorhizobium* to pH and Soil Types in the Core Cape Subregion, South Africa

**DOI:** 10.3390/genes9010002

**Published:** 2017-12-21

**Authors:** Meshack Nkosinathi Dludlu, Samson B. M. Chimphango, Charles H. Stirton, A. Muthama Muasya

**Affiliations:** Department of Biological Sciences, University of Cape Town, Private Bag X3, Rondebosch, Cape Town 7701, South Africa; Samson.Chimphango@uct.ac.za (S.B.M.C.); chstirton@gmail.com (C.H.S.); Muthama.Muasya@uct.ac.za (A.M.M.)

**Keywords:** rhizobia, *Burkholderia*, *Mesorhizobium*, Core Cape Subregion, Southern Africa, Cape Peninsula

## Abstract

Over 760 legume species occur in the ecologically-heterogeneous Core Cape Subregion (CCR) of South Africa. This study tested whether the main symbionts of CCR legumes (*Burkholderia* and *Mesorhizobium*) are phylogenetically structured by altitude, pH and soil types. Rhizobial strains were isolated from field nodules of diverse CCR legumes and sequenced for 16S ribosomic RNA (rRNA), recombinase A (*recA*) and N-acyltransferase (*nodA*). Phylogenetic analyses were performed using Bayesian and maximum likelihood techniques. Phylogenetic signals were determined using the *D* statistic for soil types and Pagel’s λ for altitude and pH. Phylogenetic relationships between symbionts of the narrowly-distributed *Indigofera superba* and those of some widespread CCR legumes were also determined. Results showed that *Burkholderia* is restricted to acidic soils, while *Mesorhizobium* occurs in both acidic and alkaline soils. Both genera showed significant phylogenetic clustering for pH and most soil types, but not for altitude. Therefore, pH and soil types influence the distribution of *Burkholderia* and *Mesorhizobium* in the CCR. All strains of *Indigofera superba* were identified as *Burkholderia*, and they were nested within various clades containing strains from outside its distribution range. It is, therefore, hypothesized that *I. superba* does not exhibit rhizobial specificity at the intragenic level. Implications for CCR legume distributions are discussed.

## 1. Introduction

There is an open debate in the microbial biogeography literature regarding whether or not microorganisms are biogeographically structured [1,2,3], thanks to the Baas Becking hypothesis that “everything is everywhere, but the environment selects” [4]. The premise of the hypothesis is that since microorganisms are small, they reproduce rapidly, they have dormancy stages and they have high dispersal potential; it follows that they should not be limited by geographical barriers and distances [5,6]. However, there is a growing body of evidence from studies on archaea, bacteria, fungi and protists, which points to the existence of microbial biogeographic structure [7,8,9,10,11,12].

Like the other microorganisms alluded to above, the various rhizobial genera exhibit some notable biogeographic structuring at local, regional, continental and global scales [13]. For example, while *Burkholderia* is the predominant symbiont of mimosoid legumes in the Brazilian Cerrado and Caatinga Biomes [14], the Mimosoid legumes occurring in Mexico are predominantly nodulated by Alphaproteobacteria, particularly the genera *Rhizobium* and *Ensifer* [15]. Genome level studies have also shown that the *Burkholderia* species that nodulate Mimosoid legumes in South America are genetically distinct from those that nodulate papilionoid legumes in the CCR of South Africa, such that they are incapable of nodulating each other’s hosts [16,17,18]. Furthermore, a recent study of the symbionts of legumes found in the sub-Himalayan region of India showed that they are nodulated by distinct *Bradyrhizobium* strains that represent new species to science [19]. Likewise, legumes of the Core Cape Subregion (CCR) of Southern Africa are predominantly nodulated by unique *Burkholderia* and *Mesorhizobium* strains [20,21,22], whereas those from the Grassland and Savannah biomes of the region are largely nodulated by unique strains of *Bradyrhizobium* [23]. Therefore, the distribution of rhizobia is as prone to biogeographic limitations as other living organisms.

Some of the factors that influence the growth and distribution of rhizobia species include pH, temperature, salinity and the distribution of suitable hosts [24,25,26,27,28,29]. These factors also affect general plant growth and nodule development [30,31]; hence, they can influence levels of nitrogen fixation. Notably, rhizobia species differ in their sensitivity to these factors. For example, species of the genus *Burkholderia* can tolerate acidic soil conditions, whereas they are replaced by alpha-rhizobia in alkaline habitats [32,33,34]. This could explain the predominance of *Burkholderia* in the acidic soils of the Cerrado, Caatinga biomes and other parts of South America [14,35], and in South Africa’s CCR, where it associates with diverse legume tribes including the Crotalarieae, Hypocalypteae, Indigofereae, Phaseoleae and Podalyrieae [20,21]. However, unlike in South America, *Burkholderia* is not the only dominant rhizobial symbiont in the CCR. *Mesorhizobium* is also an abundant symbiont, associated with a wide range of legumes in the tribes Crotalarieae, Galegeae, Genisteae and Psoralea [21,22], and the reasons for its dominance are yet to be determined.

Contrary to *Burkholderia*’s genus-wide predilection for acidic soils [34], *Mesorhizobium* species exhibit differential tolerance to environmental stress, including heavy metals, pH, salinity and temperature [27,36,37]. In terms of pH, *Mesorhizobium* species can tolerate a wide range of pH conditions (3–10), despite an optimal range of pH 6–8 [36,38]. For example, *Mesorhizobium* was found to be the dominant symbiont of *Cicer arietinum* L. (chickpea) plants growing on alkaline soils in China [39]. On the other hand, a study of *Mesorhizobium* strains nodulating chickpea plants in Portuguese soils showed that some strains were able to tolerate acidic conditions down to a minimum of pH 3 [36]. This suggests that the predominance of *Mesorhizobium* in the CCR (in addition to *Burkholderia*) might be linked to its wide-ranging tolerance to different pH conditions. Notably, while acidic soil conditions are more prevalent in the CCR, particularly in the sandstone-derived soils, patches of near neutral and alkaline soils (e.g., granite, limestone and shale) also exist [40,41]. Based on the discussion above, it appears that *Burkholderia* is more sensitive to pH, and hence, soil type, than *Mesorhizobium*. Therefore, in the case of the CCR, it is hypothesized that the distribution of *Burkholderia* species is structured by soil type and pH; while *Mesorhizobium* should be more dispersed. Moreover, *Burkholderia* should only dominate in the acidic soils, being replaced by *Mesorhizobium* species in neutral and alkaline soils.

Apart from the effects of edaphic factors on the growth and distribution of rhizobia, some studies have found correlations between turnover in the diversity of rhizobia and altitude. For example, Bontemps and co-workers [14] observed that discrete *Burkholderia* species complexes were restricted to specific altitudes in the Brazilian Caatinga and Cerrado biomes. Likewise, turnover in *Sinorhizobium* community assemblages along elevation gradients were observed in Northern China [42]. Since differences in altitude are directly related to changes in humidity and temperature [43], the correlations between altitude and rhizobial diversity suggest that rhizobial lineages vary in their sensitivity and tolerance to these attributes. The evident influence of altitudinal gradients on microbial diversity is not unique to rhizobia as similar patterns have been reported for other microorganisms, e.g., non-rhizobial bacteria and fungi [10,44,45,46]. Considering that altitude is highly variable in the CCR and that it is one of the major drivers of the diversification of the CCR flora [47], it is hypothesized that altitude influences rhizobial diversity and turnover in CCR landscapes.

Considering that the soils of the CCR are generally oligotrophic [48] and the observation that legumes have a high nitrogen-demanding lifestyle [49,50], nitrogen fixation must be a key strategy for their success in the region. Since the distribution of rhizobia is constrained by environmental factors (as previously discussed), legumes might fail to establish in habitats where their rhizobial symbionts are lacking [51,52]. Therefore, legumes that are highly specific in the kinds of rhizobia that they associate with might be restricted to habitats where their specific symbionts are present. A study by Lemaire and co-workers [21] showed that CCR legumes of the tribe Podalyrieae are exclusively nodulated by *Burkholderia* species. A subsequent study, which sampled multiple disjunct populations of the widespread *Podalyria calyptrata* Willd., found high levels of genetic diversity between the *Burkholderia* strains that nodulate the species [53]. This indicates that while *P. calyptrata* exhibits symbiotic specificity towards the genus *Burkholderia*, it associates with diverse lineages within *Burkholderia*, and this could explain its widespread distribution. Studies on the diversity of symbionts that nodulate geographically-restricted taxa are lacking for the CCR, yet such studies could shed light on the potential influence of rhizobia specificity on legume distributions. For the CCR, one such taxon is *Indigofera superba* C.H. Stirt., a rare legume species that is restricted to the Kleinrivier Mountains within the Fynbos biome of the CCR [54]. It occurs on sandstone-derived soils, at altitudes of 100–300 m [55]. It occurs in sympatry with some widespread legume species, such as *Aspalathus carnosa* Eckl. & Zeyh., *Indigofera filifolia* Thunb. and *Psoralea pullata* C.H. Stirt. Its rhizobial symbionts are presently unknown, and it is hypothesized that rhizobia specificity contributes to its limited distribution.

The main objectives of the present study were to determine if the ecological parameters; altitude, pH and soil type influence the distribution of rhizobial symbionts that nodulate various legumes of the Cape Peninsula as a microcosm of the CCR and to determine the diversity and phylogenetic position of rhizobia that associate with the narrowly-distributed *I. superba* in the CCR. The first objective was pursued through molecular characterization of rhizobial strains isolated from nodules of legume species collected in the field across the Cape Peninsula. These were analyzed together with the data from a previous study [21] that sampled broadly within the CCR. It was postulated that if an ecological parameter limits the distribution of symbionts within the landscape, then each habitat type should predominantly harbor symbionts that are suitably adapted to the local conditions. Such symbionts would likely be genetically similar. Therefore, a significant phylogenetic signal would be expected for that parameter, i.e., closely-related species would occupy similar habitats [56]. Thus, tests for phylogenetic signals for the three ecological parameters were conducted based on phylogenies of housekeeping and nodulation genes of the rhizobial strains. For the study of rhizobial symbionts of the rare *I. superba*, field nodules were sampled from multiple populations across its distribution range, and a phylogeny of its symbionts was reconstructed in a matrix that included symbionts of diverse legumes from diverse habitats within the CCR.

## 2. Materials and Methods 

### 2.1. Study Site, Nodule Sampling and Rhizobia Isolation

The primary study area was the Cape Peninsula, which is located on the south westernmost tip of the Core Cape Subregion of South Africa. Details of its climatic, edaphic, physiographic and vegetation characteristics and the selection of sampling sites are as described by Dludlu and co-workers [57]. Root nodules of legume species occurring at each site were collected and transported to the laboratory, where they were kept at 4 °C before the isolation of rhizobia, which took place within 2–5 days of sampling. Rhizobia were isolated and cultured using standard protocols [58] on yeast extract mannitol agar (YEMA), with the exception that for the surface sterilization of the nodules, a 4% solution of sodium hypochlorite (NaOCl) was used instead of acidified mercuric chloride. Rhizobial isolates were incubated at 28 °C for three to ten days depending on their growth rates, and pure cultures were obtained by sub-culturing on fresh YEMA plates. Purified cultures were suspended in 20% (*v/v*) glycerol solution and stored in a −80 °C freezer for long-term storage. This method of obtaining rhizobial cultures was chosen over the direct sequencing of DNA from the nodules because it allows to produce a pure culture that can be authenticated for nitrogen fixing properties. Furthermore, previous studies from our laboratory have shown that each nodule is occupied by a single dominant rhizobial strain [18], but nodules may be colonized by non-rhizobial bacteria.

### 2.2. DNA Extraction, Amplification and Sequencing

DNA was extracted using a modified version [59] of the cetyl trimethylammonium bromide (CTAB) DNA Extraction protocol [60]. Polymerase chain reactions (PCR) were conducted to amplify 16S ribosomic RNA (rRNA), recombinase A (*recA*) and N-acyltransferase (*nodA*) using an Applied Biosystems GeneAmp 2700 thermal cycler (Applied Biosystems, Foster City, CA, USA). Primer pairs used were 16S-f27 and 16S-r1485 [61,62] for 16S rRNA; *recA*-63F and *recA*-504R [63] for *recA*; and *nodA*-1F and *nodA*-2R [64] for *nodA*. Each PCR reaction had a total volume of 25 µL: comprising 19.92 µL of water, 2 µL of 10× buffer (Buffer A) that contained 1.5 mM Mg^2+^, 0.4 µL of 10 mM dNTP, 0.8 µL each of forward and reverse primers (10 µM), 0.08 µL of *Taq* polymerase (Kapa Biosystems, Cape Town, South Africa) and 1 µL of template DNA. All DNA regions were amplified according to the reaction conditions described by the authors of the primers, i.e. Weisburg and co-workers [61] for 16S rRNA, Gaunt and co-workers [63] for *recA* and Haukka and co-workers [64] for *nodA*. PCR products were loaded onto ethidium bromide agarose gels (1%) and subjected to electrophoresis using 0.5× Tris Borat EDTA (TBE). The gels were observed under UV light (Wavelength = 365 nm) to identify successfully amplified samples. Amplified products were enzymatically purified using the Exo/SAP protocol [65] and sent to Macrogen (Macrogen, Amsterdam, The Netherlands) for sequencing with the same primers used for PCR amplification. Newly generated sequences were deposited in the GenBank database, and the accession numbers for 16S rRNA range from MG593870–MG593941, MG704159-MG704225 for *recA* and MG704226-MG704280 for *nodA*.

### 2.3. Contig Assembly and Phylogenetic Analyses

The forward and reverse DNA sequence contigs were assembled using the Staden package Version 2.0.0 [66] and aligned using the online version of MAFFT [67]. Identification of the isolated strains was achieved by comparing individual sequences with publically available sequences on GenBank, using the Basic Local Alignment Search Tool (BLAST) of Altschul and co-workers [68]. The highest matching (% similarity) GenBank sequences for the various strains are provided as part of the supplementary materials (Table S1). The newly-generated sequences were combined with those from the study by Lemaire and co-workers [21], which sampled various legume species throughout the CCR to allow for a broader representation. The alignments were viewed in Bioedit Version 7.1.9 [69], and equivocally aligned fragments were adjusted manually. Phylogenetic analyses of the aligned matrices were performed on the Cyberinfrastructure for Phylogenetic Research (CIPRES) web portal (https://www.phylo.org), through a maximum likelihood (ML) approach, using RaxML Version 8.2.10 [70] and Bayesian inference (BI), as implemented in MrBayes Version 3.2.6 [71]. The ML analyses employed the General Time Reversible with categorized rates (GTRCAT) substitution model, and statistical support on nodes was evaluated using the non-parametric rapid bootstrapping technique [72], with 1000 replicates. For the BI analysis, the best model of nucleotide substitution was determined using jModelTest2 Version 2.1.6 [73], employing the Bayesian Information Criterion (BIC). The BI analyses were run for as many generations as necessary to achieve chain convergence (5–10 million generations). A conservative burn-in of 25% was applied to all BI analyses, and convergence of the chains was assessed using Tracer Version 1.6 [74].

To determine the combinability of the different DNA data partitions, the approach used by Pirie and co-workers [75,76] was employed. The DNA sequence data for the different genes were first analyzed separately by ML techniques as described above, and the resulting tree topologies were examined for conflicting nodes with ≥70% bootstrap support. Nodes that had <70% bootstrap support were considered unsupported, and thus, when no supported conflict was observed, the partitions were considered combinable. This approach was chosen over the widely used incongruence length difference (ILD) test [77] because the ILD only tests for overall incongruence between partitions without detecting local conflict that is due to specific taxa or clades [76]. There was no conflict observed between 16S rRNA and *recA*, and therefore, these partitions were combined in subsequent analyses. However, the *nodA* partition had significantly supported conflict with both chromosomal markers, and therefore, it was analyzed separately.

For the study of the diversity of rhizobia associated with *I. superba*, root nodules were sampled from six populations of the species across its distribution range in Vogelgat Private Nature Reserve (Hermanus, Western Cape, South Africa), sampling multiple (at least five) individuals per population to capture any potential genetic variation within and between populations. Root nodules from other legumes (i.e., *Aspalathus carnosa* Eckl. & Zeyh., *Indigofera candolleana* Meisn., *P. pullata* C.H. Stirt. and *Psoralea restioides* Eckl. & Zeyh.) that occur in the same locality as *I. superba* were also sampled to determine phylogenetic relationships between their symbionts. One chromosomal gene (*recA*) and one nodulation gene (N-acetylglucosaminyltransferase (*nodC*)) were sequenced for this study. Additional sequences from previous studies [53,78] on CCR legumes were incorporated into the dataset to determine the phylogenetic position of *I. superba* strains relative to strains nodulating other legumes in the CCR. Some sequences for reference strains, downloaded from GenBank were also included (Table S1).

### 2.4. Determination of Phylogenetic Signals

Analyses of phylogenetic signals for the various ecological parameters were conducted in R [79] using the phylogenetic trees constructed above as input and the corresponding parameters’ data as described below. Data for soil types of the sampling sites were extracted from a geological map of the CCR (shapefiles were kindly provided by the Geology Department, University of Cape Town, Western Cape, South Africa) using the site Global Positioning System (GPS) information collected during fieldwork. Soil type was coded as a binary character for each of the four soil types from which the legumes had been sampled (granite, limestone, sandstone and shale), as follows: 1, when the site belonged to a particular soil type, and 0, if it did not (Table S2). Phylogenetic structuring of rhizobial strains by soil type was tested using the *D* statistic, which measures phylogenetic signal for a discrete binary trait [80]. This was implemented using the ‘phylo.d’ function of the ‘Caper’ package, which calculates the value of *D* and tests for its significant departure from a random association and the clumping expected under a Brownian motion model [81]. The statistic *D* = 0 denotes a phylogenetically-conserved trait under a Brownian model, while *D* = 1 indicates a random distribution of traits on the tips of the phylogeny, and *D* < 0 indicates a strong phylogenetic signal, while *D* > 1 points toward phylogenetic overdispersion [80]. Significance testing was conducted using 10,000 permutations.

Altitude data for the sampled sites were recorded during field surveys using a GPS, and the soil pH was determined using the methods described by Dludlu and co-workers [57]. The raw data for altitude and pH are provided as part of the supplementary materials (Table S3). Pagel’s λ [82] was used to test for the presence of phylogenetic signal for these two continuously varying parameters. This metric ranges from 0–1, where 0 indicates that the trait evolves independently of the phylogeny and 1 indicates that the trait evolves according to the shared evolutionary history of the phylogeny’s tips, i.e., presence of phylogenetic signal [83]. The metric has proven to be robust to incomplete phylogenetic information and the presence of polytomies in the phylogenetic tree [84,85], making it suitable for the present study. The analyses were conducted using the ‘phylosig’ function of the ‘phytools’ package [86], employing 10,000 simulations for significance testing.

## 3. Results

### 3.1. Strain Identification and Phylogenetic Analyses

**Figure 1:**
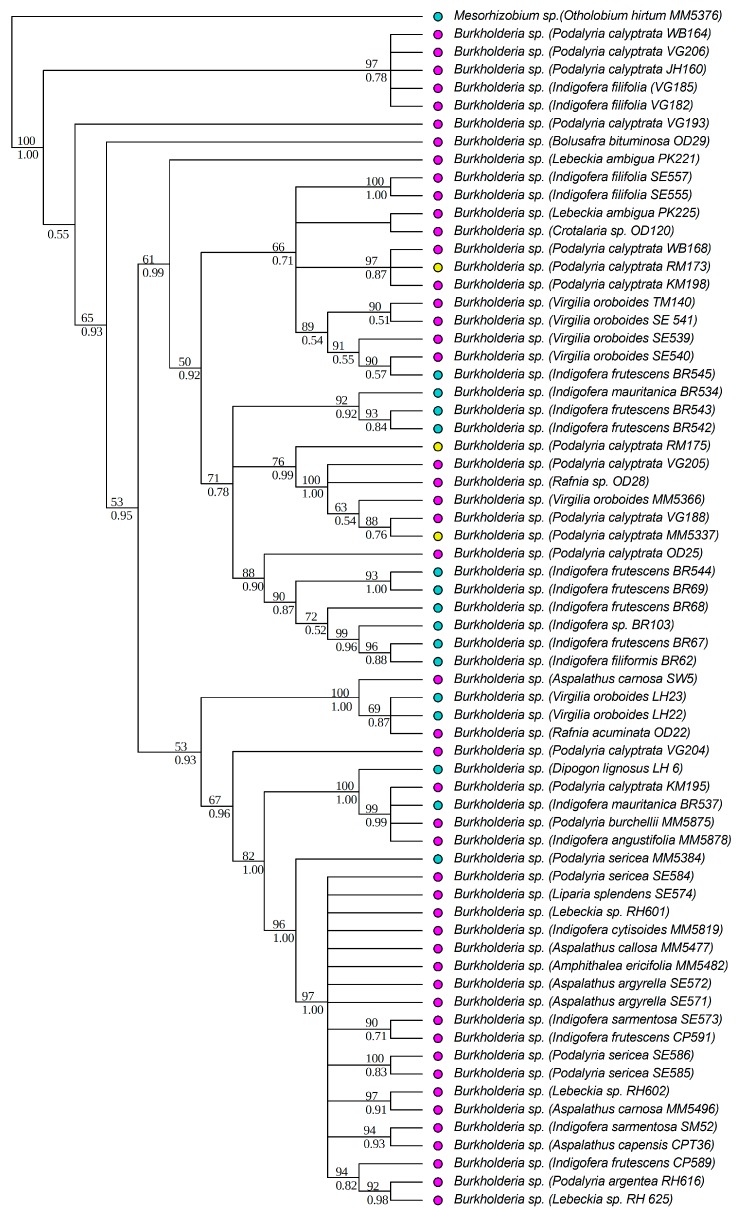
Phylogenetic relationships of *Burkholderia* strains based on 16S ribosomic RNA (rRNA) and recombinase A (*recA*) data. Names of the legume hosts and the rhizobial strain numbers are in parentheses. Strain numbers with the prefixes OD- (i.e., collector name: Oscar Dlodlo) and MM- (i.e., collector name: Muthama Muasya) are from the study by Lemaire and co-workers [21]. All other strains were newly generated in this study. Maximum likelihood (ML) bootstrap (%) and Bayesian Inference (BI) posterior probabilities are shown above and below nodes, respectively. Colored circles indicate the soil types of the sites where the legumes and their symbionts were collected, blue: granite, pink: sandstone, yellow: shale.

**Figure 2:**
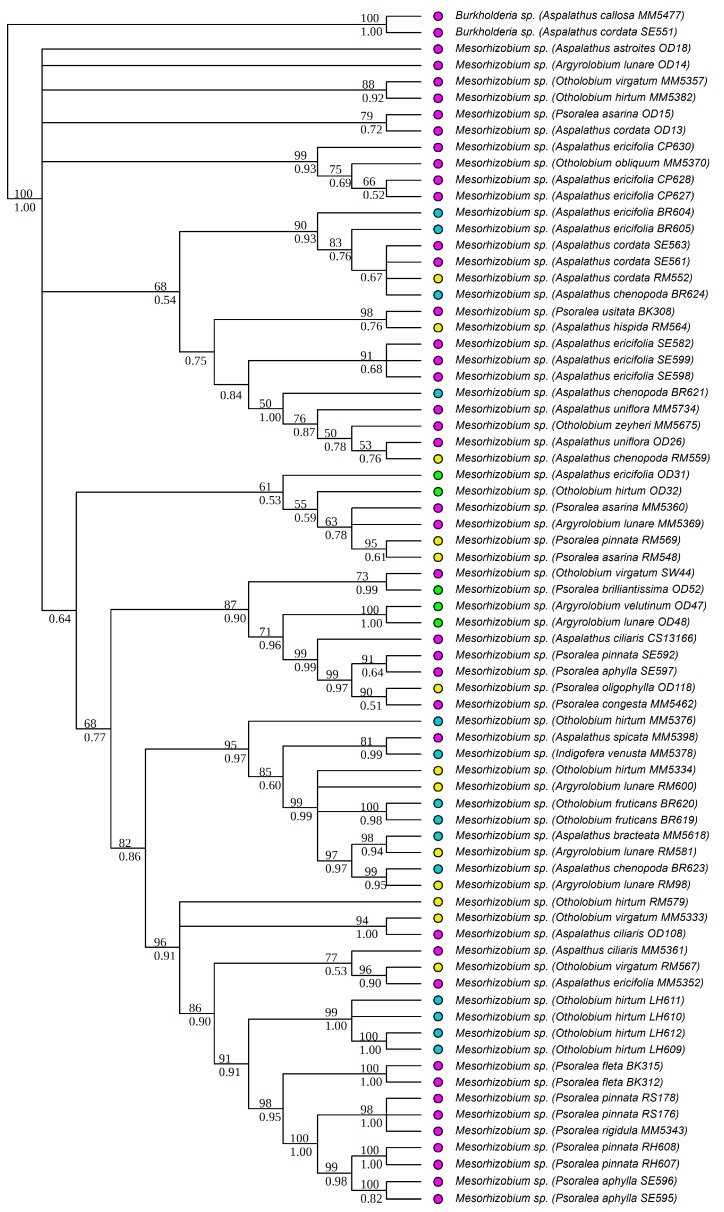
Phylogenetic relationships of *Mesorhizobium* strains based on 16S rRNA and *recA* data. Names of the legume hosts and rhizobial strain numbers are in parentheses. Strain numbers with the prefixes OD- and MM- are from the study by Lemaire and co-workers [21]. The rest were newly generated in this study. ML bootstrap (%) and BI posterior probabilities are shown above and below nodes, respectively. Colored circles indicate the soil types of the sites where the legumes and their symbionts were collected, blue: granite; green: limestone; pink: sandstone, yellow: shale.

**Figure 3:**
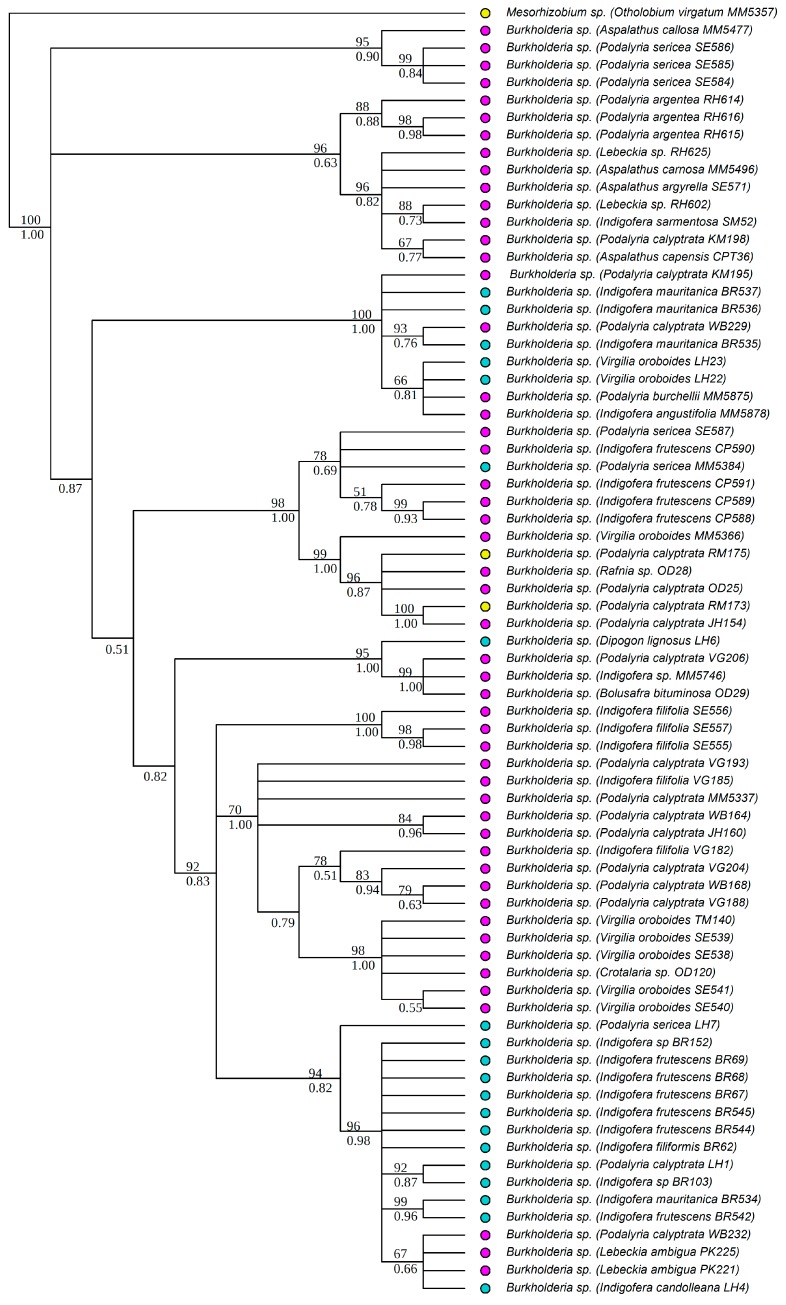
Phylogenetic relationships of *Burkholderia* strains based on N-acyltransferase (*nodA*) data. Names of the legume hosts and rhizobial strain numbers are in parentheses. Strain numbers with the prefixes OD- and MM- are from the study by Lemaire and co-workers [21]. The rest were newly generated in this study. ML bootstrap (%) and BI posterior probabilities are shown above and below nodes, respectively. Colored circles indicate the soil types of the sites where the legumes and their symbionts were collected, blue: granite; pink: sandstone, yellow: shale.

**Figure 4:**
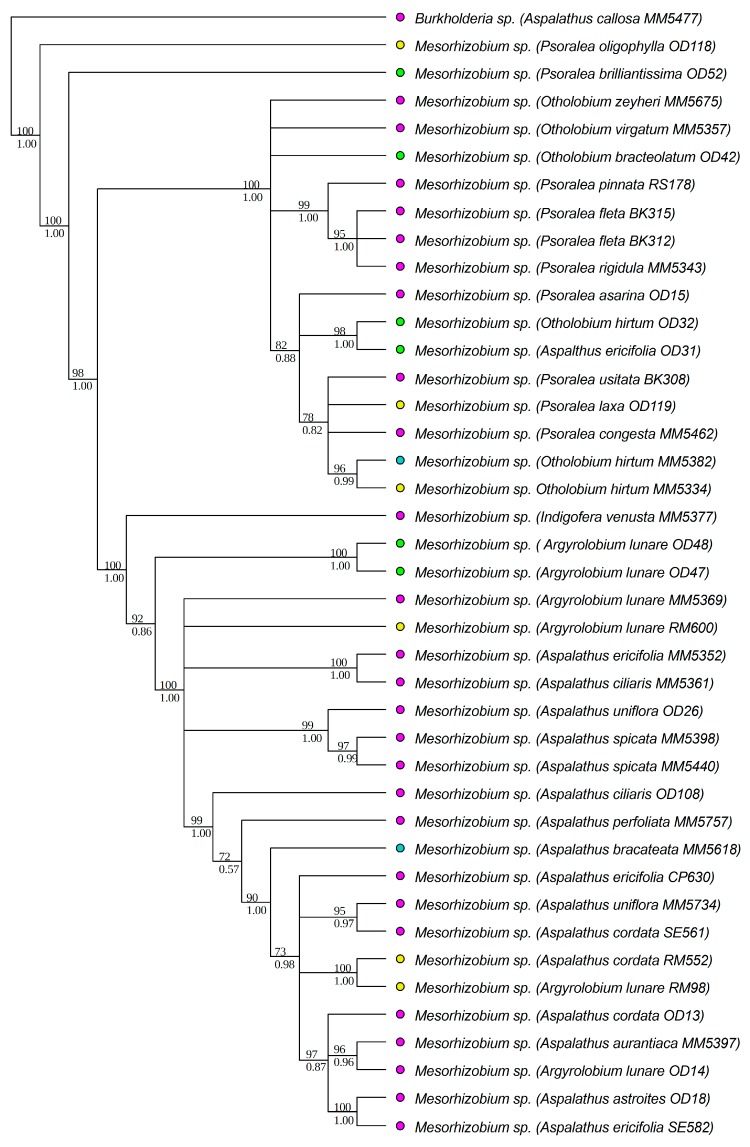
Phylogenetic relationships of *Mesorhizobium* strains based on *nodA* data. Names of the legume hosts and rhizobial strain numbers are in parentheses. Strain numbers with the prefixes OD- and MM- are from the study by Lemaire and co-workers [21]. The rest were newly generated in this study. ML bootstrap (%) and BI posterior probabilities are shown above and below nodes, respectively. Colored circles indicate the soil types of the sites where the legumes and their symbionts were collected, blue: granite; green: limestone; pink: sandstone, yellow: shale.

All strains isolated as part of this study were identified to the genus level, based on BLAST [68] search results of individual sequences, as belonging to either *Burkholderia* or *Mesorhizobium*. All *Burkholderia* strains had at least 97% similarity to known South African strains, while the *Mesorhizobium* strains were similar to rhizobial strains from various parts of the world. Strains isolated from the legume genera *Aspalathus* L. (except for *Aspalathus callosa*, *Aspalathus capensis* and *A. carnosa*), *Argyrolobium* Eckl. & Zeyh., *Otholobium* C.H. Stirt. and *Psoralea* L. were identified as *Mesorhizobium*. *Burkholderia* strains were from *Amphithalea* Eckl. & Zeyh., *Aspalathus* L., *Bolusafra* Kuntze., *Dipogon* Liebm., *Indigofera* L., *Lebeckia* Thunb., *Podalyria* Willd., *Rafnia* Thunb. and *Virgilia* Poir. Phylogenetic analyses were conducted separately for each of the two genera to allow for independent analyses of phylogenetic signals within each genus. The aligned 16S rRNA matrix of *Burkholderia* consisted of 67 strains and 1540 characters (total aligned length), while that of *Mesorhizobium* had 73 strains and 1520 characters. The *recA* matrix for *Burkholderia* had 67 strains and 951 characters, while that of *Mesorhizobium* had 67 strains and 886 characters. The Bayesian and ML analyses of the individual chromosomal gene regions produced trees of similar topologies, and in all cases, the *recA* tree was better resolved than that of the 16S rRNA. The trees from the concatenated matrices were better resolved and more strongly supported (Figure 1 and Figure 2) than the individual gene trees. The aligned *nodA* matrix for *Burkholderia* had 74 rhizobial strains and 734 characters, while that of *Mesorhizobium* had 41 strains and 674 characters. The Bayesian and ML trees had similar topologies and were well supported (Figure 3 and Figure 4). However, for both *Burkholderia* and *Mesorhizobium*, the *nodA* topologies were incongruent to those of the chromosomal gene trees, suggesting disparate evolutionary histories between the chromosomal and nodulation genes.

### 3.2. Analyses of Phylogenetic Signals

For *Burkholderia*, 72% of the strains occurred on sandstone, 24% on granite, 4% on shale and none were on limestone-derived soils, whereas for *Mesorhizobium*, 54% of the strains were on sandstone, 20% on granite, 17% on shale and 9% on limestone-derived soils (Table S2). From the chromosomal gene tree, a comparison of the phylogenetic *D* statistic with the random shuffling of parameter values along the tips of the phylogeny showed a significant phylogenetic signal for sandstone (*D* = 0.133; *p* = 0.00) and a strong phylogenetic signal for granite (*D* = −0.22; *p* = 0.00) for *Burkholderia*. *Mesorhizobium* had significant phylogenetic signals for sandstone (*D* = 0.433; *p* = 0.0009) and granite (*D* = 0.252; *p* = 0.0006) and a strong phylogenetic signal for limestone-derived (*D* = −0.359; *p* = 0.0006) soils (Table 1). On the other hand, when the *D* statistic was compared to the Brownian threshold model, all but the *Mesorhizobium* on shale-derived soils were as clumped on the phylogeny as expected under a Brownian motion model (Table 1).

For the *nodA* phylogeny, *Burkholderia* showed similar patterns of phylogenetic signal as observed for the chromosomal genes (Table 1). Despite having similar patterns to those of the chromosomal genes for sandstone and limestone-derived soils, *nodA* results for *Mesorhizobium* showed evidence of random dispersion of parameter values for granite-derived soils (*D* = 1.752; *p* = 0.833), with a significant departure (*p* = 0.032) from a Brownian threshold model (Table 1), indicating a lack of phylogenetic structure for this parameter.

Analyses based on the chromosomal gene trees showed significant phylogenetic signals for pH for both *Burkholderia* (Pagel’s λ = 0.642; *p* = 0.019) and *Mesorhizobium* (Pagel’s λ = 0.508; *p* = 0.027). On the other hand, altitude showed no significant phylogenetic signal on either *Burkholderia* (Pagel’s λ = 0.402; *p* = 0.081) or *Mesorhizobium* (Pagel’s λ = 0.217; *p* = 0.097). Similar patterns were observed for analyses based on the *nodA* trees of both rhizobial genera (Table 2).

### 3.3. Diversity of Rhizobial Symbionts of Indigofera superba

**Figure 5:**
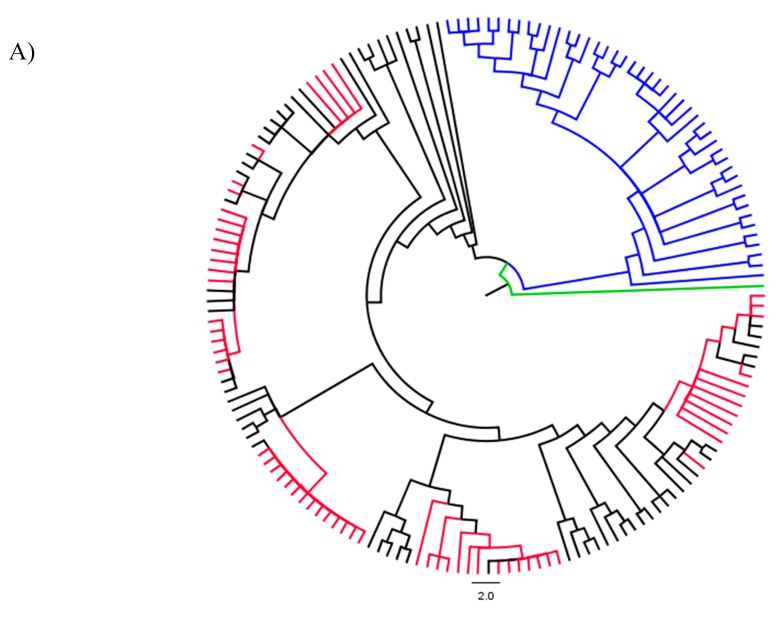

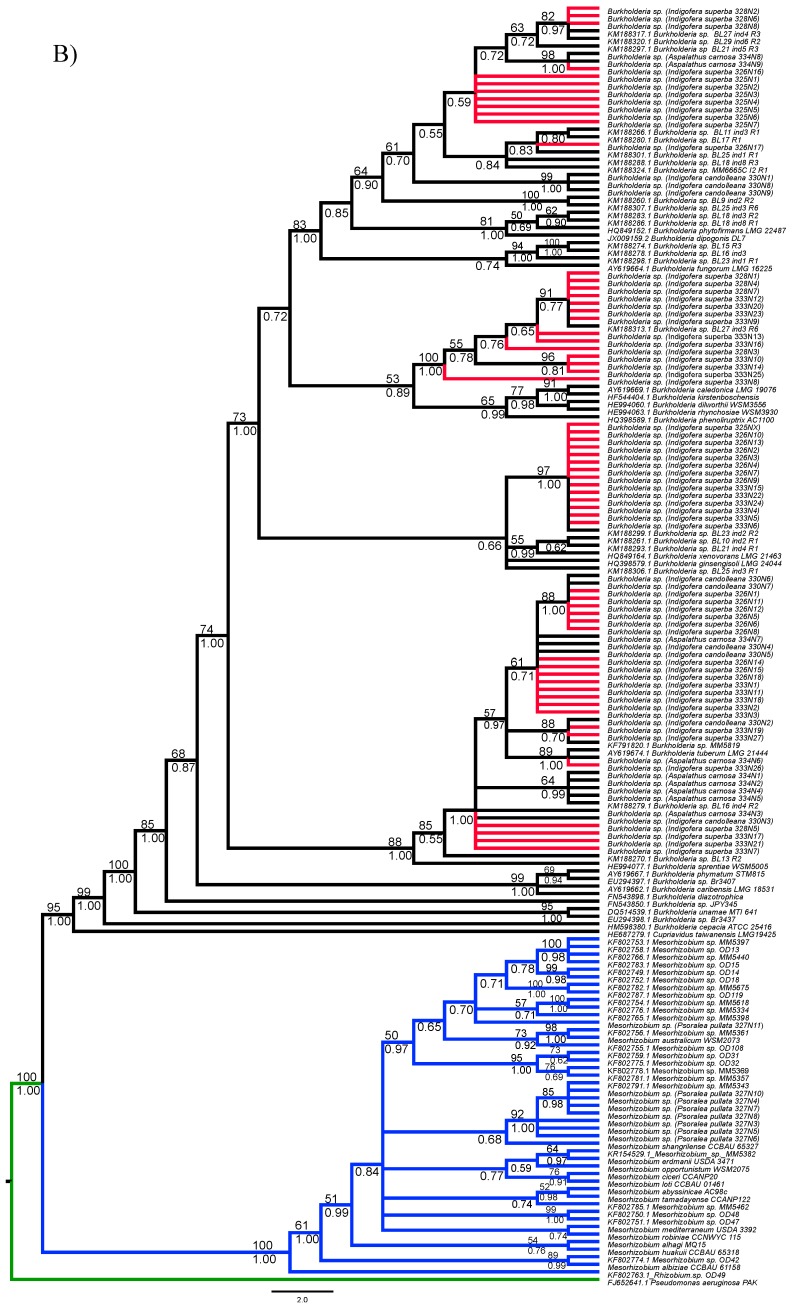
(**A**) Phylogenetic relationships of rhizobial strains based on *recA* data, showing the phylogenetic position of strains isolated from *Indigofera superba* (red nodes) in relation to rhizobial strains of other legumes in the CCR, blue: *Mesorhizobium* strains; black: *Burkholderia*, green: outgroup. (**B**) Detailed version of Figure 5A), showing the tip labels and ML Bootstrap support values (%) above the nodes and Bayesian posterior probabilities, below the branches.

**Figure 6:**
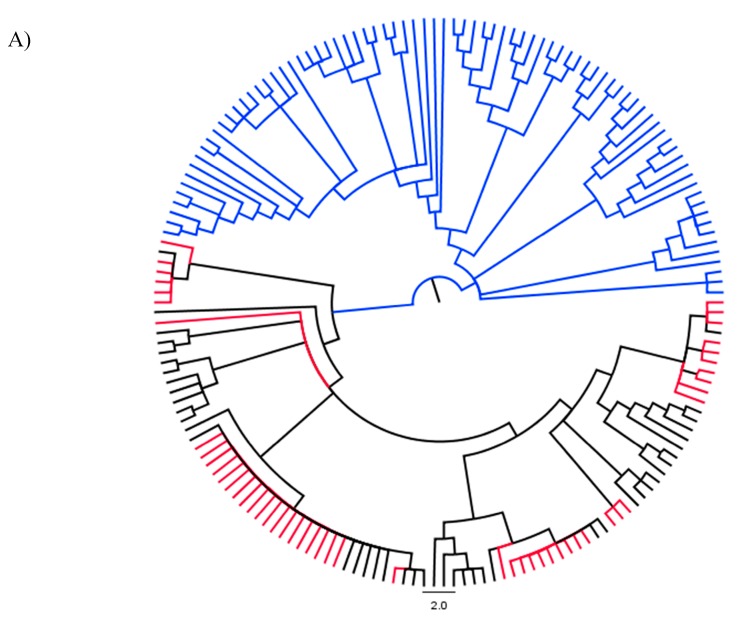

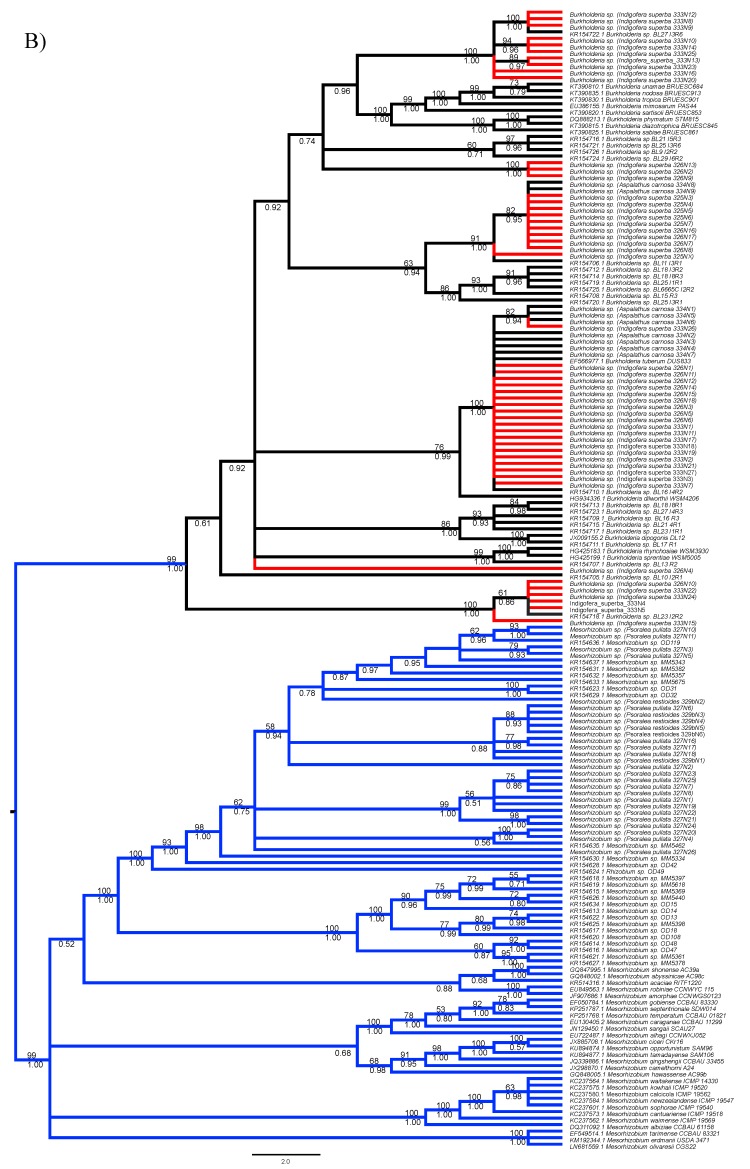
(**A**) Phylogenetic relationships of rhizobial strains based on *nodC* data, showing the phylogenetic position of strains isolated from *I. superba* (red nodes) in relation to rhizobial strains of other legumes in the CCR, blue: *Mesorhizobium* strains; and black: *Burkholderia* strains. (**B**) Detailed version of Figure 6A), showing the tip labels and ML and Bootstrap support values (%) above branches and Bayesian posterior probabilities below branches.

In total, 87 and 86 strains were isolated and successfully sequenced for *recA* and *nodC*, respectively, from *I. superba* and its sympatric legumes in Vogelgat Private Nature Reserve (South Africa). All strains that were isolated from the root nodules of *I. superba* were identified (based on BLASTn searches on GenBank) as *Burkholderia*, and the highest matches ( ≥ 95% similarity) were known *Burkholderia* species from South Africa, i.e., *Burkholderia dilworthii*, *Burkholderia kirstenboschensis*, *Burkholderia rhynchosiae*, *Burkholderia sprentiae* and *Burkholderia tuberum*. The highest matching (% similarity) GenBank sequences for the various strains are provided as part of the supplementary materials (Table S1). On the other hand, all strains isolated from *P. pullata*, which occurs in sympatry with *I. superba*, were *Mesorhizobium*. The BI and ML analyses of the *recA* and *nodC* matrices produced some fairly resolved and supported topologies (Figure 5 and Figure 6). *Indigofera superba* symbionts were part of multiple distinct clades, most of which included strains isolated from other legume species that occur outside its distribution range in the CCR in both the *recA* and the *nodC* trees (Figure 5 and Figure 6).

## 4. Discussion

The main objective of this study was to determine if the three ecological parameters, altitude, pH and soil type, show phylogenetic structuring for the two predominant rhizobial genera, *Burkholderia* and *Mesorhizobium* [21], in the Core Cape Subregion of South Africa. For both genera, the results showed significant phylogenetic signals for soil type and pH, but not for altitude. Soil type can be viewed as an indicator of the nutrient status of the habitats based on the literature [87,88] and on the results of Dludlu and co-workers [57], which showed that sandstone habitats are the most nutrient-impoverished relative to the granite and shale substrates. Limestone soils are generally more fertile than the granite, sandstone and shale substrates [48,89]. Soil type is also related to pH, with the following general ranges, sandstone pH: 3–4.5, granite pH: 4.5–5.5, shale pH: 5.5–6.5 and limestone pH: > 6.5 [48,88,90]. Therefore, it is unsurprising that the results show similar patterns for pH and soil type.

Consistent with previous studies [14,34], *Burkholderia* strains showed a preference for acidic soils, as indicated by the large proportion (72%) of its strains that were collected from the highly acidic sandstone habitats and its complete absence in the limestone habitats, which have alkaline conditions (Table S2). The findings of significant phylogenetic signals on the acidic sandstone and granite habitats indicate that in addition to the genus-wide preference for acidic conditions, *Burkholderia* strains are not randomly distributed within these soil types, but closely-related strains tend to occupy similar habitats with respect to soil type and pH. Thus, these ecological parameters have a significant influence on *Burkholderia*’s distribution within the CCR landscape. On the other hand, the results indicated that *Mesorhizobium* tolerates a wider range of soil types and pH conditions because it had nearly equal proportions of its strains isolated from the highly acidic and infertile sandstones and the higher pH and nutrient rich substrates (Table S2). The finding of significant phylogenetic signals for granite, limestone and sandstone and for pH indicates that despite the wider tolerance range of *Mesorhizobium* as a genus, the distribution of various strains is phylogenetically structured. Thus, for each of the different soil types and pH conditions of the CCR, there are particular strains of *Mesorhizobium* that are adapted to them. This is consistent with observations from other biomes showing that *Mesorhizobium* species exhibit high diversity in their tolerance to various pH conditions [27,37,91]. This could explain the predominance of *Mesorhizobium* (in addition to *Burkholderia*) in the CCR [21]. Overall, the results suggest that *Mesorhizobium* has a wider soil type and pH tolerance range than *Burkholderia*, and strains of both genera exhibit phylogenetic clustering within their distribution ranges.

The finding of a significant phylogenetic signal for granite-derived soils (for *Mesorhizobium*) based on the chromosomal gene tree, versus a lack of phylogenetic signal for the same parameter on the *nodA* tree suggests that the chromosomal and nodulation genes have different evolutionary histories, possibly due to horizontal inheritance of the nodulation genes. This would be unsurprising as studies [20,78] show that horizontal gene transfer (HGT) is a common phenomenon among CCR rhizobia, leading to conflicting phylogenetic signals between chromosomal and nodulation genes.

The observed variation in the biogeographical structuring of the different rhizobia with respect to soil type and pH has implications for the biogeography of legumes in the CCR. This is particularly the case considering that distinct edaphic habitats are characterized by discrete legume assemblages in the Cape Peninsula [57], which points to an important role of edaphic factors in driving legume biogeography. Considering that soil nutrients are a limiting factor to plants in the CCR [48], the ecological advantage that nitrogen fixation confers on legumes must be key to their success in such an environment. Therefore, if edaphic factors also limit the distribution of rhizobia, legumes that exhibit high rhizobial specificity, e.g., species of the tribe Podalyrieae, which are only nodulated by *Burkholderia* [21], might fail to establish in habitats that are unsuitable for their rhizobial symbionts [51,52]. In such a case, the biogeography of such legumes would also be driven by the distribution of their specific symbionts. This could explain the sparse representation of the tribe Podalyrieae in the limestone habitats (three out of 104 species), whereas most of its species occur in sandstone habitats (i.e., where *Burkholderia* are the predominant symbionts) in the CCR [92]. On the contrary, promiscuous legume lineages, e.g., *Aspalathus* and *Indigofera*, or those that are nodulated by *Mesorhizobium*, e.g., *Psoralea* and *Otholobium* [21], are widespread in diverse soil types of the CCR [92]. These patterns suggest that rhizobia play a significant role in the distribution of legumes in the CCR. Furthermore, in a glasshouse experiment where legumes from the Fynbos and Grassland biomes were grown in soils from both biomes, Fynbos legumes were only able to nodulate in Fynbos soil (Lemaire and co-workers, unpublished [93]). This indicates a potential role of rhizobia specificity in driving the distribution of legumes in the various biomes of Southern Africa. This, therefore, opens up avenues for further research. For example, can rhizobia specificity explain why some Cape clades that occur outside the Fynbos are restricted to sandstone habitats? Furthermore, why are genistoid legumes that occur in the CCR nodulated by *Mesorhizobium* [21], whereas those of the Great Escarpment are nodulated by *Bradyrhizobium*? [23].

Although altitude is highly heterogeneous and it has been found to play a significant role in driving plant diversification in the CCR [47], the results of the present study showed no evidence of phylogenetic structuring of the two predominant rhizobial genera, for this ecological parameter. Similar results were obtained in the study conducted by Lemaire and co-workers [21] for both the chromosomal (16S rRNA) and *nodA* genes of *Mesorhizobium*. Likewise, the *nodA* genetic diversity in *Burkholderia* was not significantly correlated with altitude [21]. The only disparity is that they [21] found a positive correlation between altitude and genetic diversity of 16S rRNA for *Burkholderia*. The disparity is likely because the current study considered overall phylogenetic signals based on a combination of both 16S rRNA and *recA*, whereas the previous study only considered genetic distances of a single chromosomal marker: 16S rRNA. These findings are contrary to the observed biogeographic structuring of *Burkholderia* communities along altitudinal gradients in Brazil [14]. Such conflicting results have also been observed in other rhizobial genera, e.g., *Sinorhizobium* in Northern China, where the diversity of nodule isolates from different sites was correlated with altitude [42], versus central China, where they found no correlation between altitude and the genetic variation of rhizobial strains cultured from soils collected from sites of different altitudes [94]. Although the latter study was conducted under glasshouse conditions while the former was based on field nodules, the soils used for the trapping experiments were from sites of different altitudes [94], which validates comparing the two studies. Considering that the glasshouse trapping experiments were conducted under the same conditions for all the different soils, changes in rhizobial diversity (if any) as a result of the glasshouse conditions would have to be homogeneous across the samples. However, the sampling of the present study and that of Lemaire and co-workers [21] spanned an altitude range of 10–1000 m above sea level, whereas the highest altitude in the CCR is 2249 m [47]. Thus, the current data may not be sufficient to allow for conclusive inferences on the role of altitude in rhizobial biogeography for the region. Hence, future studies, sampling higher altitude areas could allow for further investigation of the effect of altitude on rhizobia diversity and turnover in the CCR.

The finding that all strains isolated from the root nodules of the rare *I. superba* belong to the genus *Burkholderia* (despite the availability of *Mesorhizobium*, which was isolated from its sympatric species, *P. pullata*) suggests a potential symbiotic specificity at the generic level. However, the dispersion of the different strains in several distinct clades points towards association with multiple divergent lineages within the genus *Burkholderia*. If these strains that cluster with divergent lineages are capable of nodulating *I. superba*, it would be a similar scenario to that of the widespread *P. calyptrata*, which is nodulated by strains from several distinct lineages within *Burkholderia* [53], i.e., no symbiotic specificity at the intragenic level. The results also suggest that the strains isolated from *I. superba* are not genetically distinct since they were part of various clades that included strains isolated from legumes that occur outside its distribution range. Overall, these results lead to the hypothesis that *I. superba* does not exhibit rhizobia specificity at the intragenic level. More studies are required to test this hypothesis, and this could involve testing if the various strains are able to induce nodulation on *I. superba* and determining if *I. superba* is able to form nodules in soils from outside its distribution range. A lack of nodulation from these soils would indicate that the restricted distribution of *I. superba* is due to rhizobia specificity.

## 5. Conclusions

The study of the legume-rhizobia relationship in Southern Africa is still at its infancy, and although the CCR has received more attention relative to the rest of the sub-continent, only a small proportion of its legume diversity has been studied. Nevertheless, the patterns that are emerging from these few studies suggest that these below ground mutualists of the legumes might be significant drivers of the distribution of legumes in the CCR. The findings of the present study suggest that while *Burkholderia* has an affinity for the acidic and nutrient-poor soils of the CCR, *Mesorhizobium* has a wider soil type and pH tolerance range, allowing various strains to thrive in habitats of varying edaphic stress. The presence of such ecologically diverse symbionts, coupled with the edaphic heterogeneity of the CCR landscape provide opportunities for the legumes to diversify, and this might explain the high species richness of the family. With the finding that rhizobia contribute towards the structuring of legume assemblages in the CCR, it is plausible that rhizobia also have a strong influence on the structuring of legume assemblages within and across the different biomes of Southern Africa, and this provides a potential direction for future research.

## Figures and Tables

**Table 1:** Results of the tests of phylogenetic signals on soil types using the *D* statistic on the combined chromosomal gene (16S ribosomic RNA (rRNA) and recombinase A (*recA*)), and the N-acyltransferase (*nodA*) trees of *Burkholderia* and *Mesorhizobium.*

DNA Region	Genus	Soil Type	*D*	*p*-Value Random Shuffle	*p*-Value Brownian Motion
16S rRNA and *recA*	*Burkholderia*	Granite Sandstone Shale	−0.220 0.133 0.956	0.00 0.00 0.375	0.717 0.384 0.144
	*Mesorhizobium*	Granite Limestone Sandstone Shale	0.252 −0.359 0.133 0.975	0.0006 0.0006 0.0009 0.419	0.235 0.744 0.056 0.001
*nodA*	*Burkholderia*	Granite Sandstone Shale	−0.208 0.082 0.617	0.00 0.00 0.160	0.715 0.424 0.291
	*Mesorhizobium*	Granite Limestone Sandstone shale	1.752 −0.871 0.162 0.492	0.833 0.0005 0.002 0.118	0.032 0.914 0.423 0.218

**Table 2:** Results of the tests of phylogenetic signals for altitude and pH using Pagel’s λ on the chromosomal (16S rRNA and *recA*) and *nodA* trees of *Burkholderia* and *Mesorhizobium.*

Genus	Gene Type	Variable	Pagel’s λ	*p*-Values
*Burkholderia*	Chromosomal	Altitude pH	0.402 0.643	0.081 0.019
	*nodA*	Altitude pH	0.093 0.840	0.389 0.0008
*Mesorhizobium*	Chromosomal	Altitude pH	0.217 0.508	0.097 0.027
	*nodA*	Altitude pH	0.767 0.912	0.999 0.016

## Supplementary Materials

The following are available online at www.mdpi.com/2073-4425/9/1/2/s1. Table S1: BLASTn search results for the various rhizobial strains isolated for this study, Table S2: List of rhizobial strains with binary scoring of their soil types, Table S3: List of rhizobial strains used with the altitude and pH data of their sites.
