# Supplementary material for: Differential Preference of Burkholderia and Mesorhizobium to pH and Soil Types in the Core Cape Subregion, South Africa
Source: Genes (Basel). 2017 Dec 21;9(1):2. doi: 10.3390/genes9010002 (PMC5793155; doi:10.3390/genes9010002)
Supplement: Supplementary file 1 [file genes-09-00002-s001.pdf]

Supplementary Materials

Table S1. BLASTn search results for the various rhizobial strains isolated as part of this study. Percentage similarity to their closest sequences on GenBank are shown.

DNA region	Strain number	Legume host	Closest match (GenBank Accession No.)	% Similarity
16S rRNA	RM581	*Argyrolobium lunare*	*Mesorhizobium sp. CCBAU 33406 (JQ339800.1)*	95
	RM600	*Argyrolobium lunare*	*Mesorhizobium sp. strain WC23.1d (MG050062.1)*	99
	RM98	*Argyrolobium lunare*	*Mesorhizobium sp. strain WC23.1d (MG050062.1)*	100
	SE571	*Aspalathus argyrella*	*Burkholderia sp. WSM4177 (HE862275.1)*	97
	SE572	*Aspalathus argyrella*	*Burkholderia sp. HC6.4b (HF674718.1)*	99
	CPT36	*Aspalathus capensis*	*Burkholderia sp. strain WC21.1b (MG050053.1)*	97
	SW5	*Aspalathus carnosa*	*Burkholderia sp. WK1.1f (HF674688.1)*	99
	BR621	*Aspalathus chenopoda*	*Mesorhizobium sp. strain WC23.1d (MG050062.1)*	98
	BR624	*Aspalathus chenopoda*	*Mesorhizobium sp. strain WC23.1d (MG050062.1)*	96
	RM552	*Aspalathus cordata*	*Mesorhizobium loti NZP2037 (CP016079.1)*	99
	SE551	*Aspalathus cordata*	*Burkholderia sp. BL25 (KR154605.1)*	97
	SE563	*Aspalathus cordata*	*Mesorhizobium sp. CCBAU 33404 (JQ339778.1)*	99
	BR604	*Aspalathus ericifolia*	*Mesorhizobium sp. CCBAU 11214 (EF149002.1)*	100
	BR605	*Aspalathus ericifolia*	*Mesorhizobium loti NZP2037 (CP016079.1)*	99
	CP627	*Aspalathus ericifolia*	*Mesorhizobium sp. RPJ3 (FJ985026.1)*	97
	CP630	*Aspalathus ericifolia*	*Mesorhizobium sp. strain WC23.1d (MG050062.1)*	99
	LH6	*Dipogon lignosus*	*Burkholderia sp. WSM4178 (HE862279.1)*	99
	SE555	*Indigofera filifolia*	*Burkholderia sp. CS13775 (KF791624.1)*	97
	SE557	*Indigofera filifolia*	*Burkholderia sp. CS 2 (AY178065.1)*	99
	VG182	*Indigofera filifolia*	*Burkholderia sp. BL16 R3 (KR154595.1)*	99
	VG185	*Indigofera filifolia*	*Burkholderia sp. BL16 R3 (KR154595.1)*	99
	BR62	*Indigofera filiformis*	*Burkholderia sp. RAU2i (HF674680.1)*	97
	BR542	*Indigofera frutescens*	*Burkholderia sp. RAU2i (HF674680.1)*	99
	BR543	*Indigofera frutescens*	*Burkholderia sp. BL6665C (KR154611.1)*	98

DNA region	Strain number	Legume host	Closest match (GenBank Accession No.)	% Similarity
	BR544	*Indigofera frutescens*	*Burkholderia sp. BL6665C (KR154611.1)*	98
	BR545	*Indigofera frutescens*	*Burkholderia sp. BL25 (KR154605.1)*	97
	BR67	*Indigofera frutescens*	*Burkholderia sp. RAU2i (HF674680.1)*	99
	BR68	*Indigofera frutescens*	*Burkholderia sp. RAU2i (HF674680.1)*	99
	BR69	*Indigofera frutescens*	*Burkholderia sp. RAU2i (HF674680.1)*	98
	CP589	*Indigofera frutescens*	*Burkholderia sp. WSM4177 (HE862275.1)*	98
	CP591	*Indigofera frutescens*	*Burkholderia sp. UCT 31 (AY178074.1)*	98
	BR537	*Indigofera mauritanica*	*Burkholderia sp. RAU2j (HF674681.1)*	98
	SE573	*Indigofera sarmentosa*	*Burkholderia sp. Cpub 6 (AY178071.1)*	99
	SM52	*Indigofera sarmentosa*	*Burkholderia sp. WSM4177 (HE862275.1)*	98
	BR103	*Indigofera sp.*	*Burkholderia sp. RAU2i (HF674680.1)*	99
	PK221	*lebeckia ambigua*	*Burkholderia sp. RAU2i (HF674680.1)*	99
	PK225	*lebeckia ambigua*	*Burkholderia sp. BL25 (KR154605.1)*	100
	RH601	*Lebeckia sp.*	*Burkholderia sp. HC6.4b (HF674718.1)*	99
	RH602	*Lebeckia sp.*	*Burkholderia sp. Cpub 6 (AY178071.1)*	99
	RH625	*Lebeckia sp.*	*Burkholderia sp. WSM4177 (HE862275.1)*	99
	SE574	*Liparia splendens*	*Burkholderia sp. HC6.4b (HF674718.1)*	99
	BR619	*Otholobium fruticans*	*Mesorhizobium sp. MM5377 (KF802585.1)*	99
	LH610	*Otholobium hirtum*	*Mesorhizobium cantuariense strain ICMP 19515 (NR_137373.1)*	99
	LH611	*Otholobium hirtum*	*Mesorhizobium ciceri strain VAR5.8 (KY515337.1)*	100
	LH612	*Otholobium hirtum*	*Mesorhizobium ciceri strain VAR5.8 (KY515337.1)*	100
	RM579	*Otholobium hirtum*	*Mesorhizobium sp. strain WC33b*	98
	RH616	*Podalyria argentea*	*Burkholderia sp. WSM4177 (HE862275.1)*	99
	JH160	*Podalyria calyptrata*	*Burkholderia sp. BL16 R3 (KR154595.1)*	99
	KM195	*Podalyria calyptrata*	*Burkholderia sp. RAU2j (HF674681.1)*	99
	RM173	*Podalyria calyptrata*	*Burkholderia sp. BL6665C (KR154611.1)*	99
	RM175	*Podalyria calyptrata*	*Burkholderia sp. WK1.1f (HF674688.1)*	99
	VG193	*Podalyria calyptrata*	*Burkholderia sp. BL16 R3 (KR154595.1)*	99
	VG204	*Podalyria calyptrata*	*Burkholderia sp. RAU2i (HF674680.1)*	99

DNA region	Strain number	Legume host	Closest match (GenBank Accession No.)	% Similarity
	VG206	*Podalyria calyptrata*	*Burkholderia sp. BL16 R3 (KR154595.1)*	97
	WB164	*Podalyria calyptrata*	*Burkholderia sp. RAU2f (HF674677.2)*	99
	WB168	*Podalyria calyptrata*	*Burkholderia sp. BL29 I6R2 (KR154610.1)*	99
	SE584	*Podalyria sericea*	*Burkholderia sp. HC6.4b (HF674718.1)*	99
	SE596	*Psoralea aphylla*	*Mesorhizobium sp. strain WSM4692 (MF949006.1)*	99
	SE597	*Psoralea aphylla*	*Mesorhizobium sp. ORS1080 (AJ295082.1)*	99
	RM548	*Psoralea asarina*	*Mesorhizobium sp. CCBAU 41174 (KP116972.1)*	99
	BK312	*Psoralea fleta*	*Mesorhizobium sp. strain WSM4692 (MF949006.1)*	99
	BK315	*Psoralea fleta*	*Mesorhizobium sp. strain WSM4692 (MF949006.1)*	99
	RH607	*Psoralea pinnata*	*Mesorhizobium sp. strain WSM4692 (MF949006.1)*	98
	RM569	*Psoralea pinnata*	*Mesorhizobium sp. CCBAU 41174 (KP116972.1)*	99
	RS176	*Psoralea pinnata*	*Mesorhizobium sp. strain WSM4692 (MF949006.1)*	100
	RS178	*Psoralea pinnata*	*Mesorhizobium sp. strain WSM4692 (MF949006.1)*	100
	SE592	*Psoralea pinnata*	*Mesorhizobium sp. ORS1080 (AJ295082.1)*	100
	BK308	*Psoralea usitata*	*Mesorhizobium loti NZP2037 (CP016079.1)*	100
	LH22	*Virgilia oroboides*	*Burkholderia kirstenboschensis HC1.1bc (HF674714.1)*	99
	LH23	*Virgilia oroboides*	*Burkholderia kirstenboschensis HC1.1bc (HF674714.1)*	99
	SE539	*Virgilia oroboides*	*Burkholderia sp. CB2 (AY178059.1)*	99
	SE540	*Virgilia oroboides*	*Burkholderia sp. CB2 (AY178059.1)*	98
nodA	SE571	*Aspalathus argyrella*	*Burkholderia tuberum strain DUS833 (EF566976.1)*	99
	CPT36	*Aspalathus capensis*	*Burkholderia tuberum strain DUS833 (EF566976.1)*	99
	LH6	*Dipogon lignosus*	*Burkholderia rhynchosiae strain WSM3930 (EU219866.1)*	100
	LH4	*Indigofera candolleana*	*Paraburkholderia sprentiae WSM5005 (CP017562.1)*	98
	SE555	*Indigofera filifolia*	*Burkholderia sp. BL21-ind2-R1 (KM188382.1)*	100
	SE556	*Indigofera filifolia*	*Burkholderia sp. BL21-ind4-R3 (KM188386.1)*	99
	SE557	*Indigofera filifolia*	*Burkholderia sp. BL21-ind2-R1 (KM188382.1)*	100
	VG182	*Indigofera filifolia*	*Burkholderia sp. BL16-ind1-R2 (KM188366.1)*	100
	VG185	*Indigofera filifolia*	*Burkholderia sp. BL16-ind1-R2 (KM188366.1)*	99
	BR62	*Indigofera*	*Burkholderia sp. MM6669-R2 (KM188348.1)*	100

DNA region	Strain number	Legume host	Closest match (GenBank Accession No.)	% Similarity
		filiformis		
	BR542	*Indigofera frutescens*	*Burkholderia sp. MM6669-R2 (KM188348.1)*	99
	BR544	*Indigofera frutescens*	*Burkholderia sp. MM6669-R2 (KM188348.1)*	100
	BR545	*Indigofera frutescens*	*Burkholderia sp. MM6669-R2 (KM188348.1)*	100
	BR67	*Indigofera frutescens*	*Burkholderia sp. MM6669-R2 (KM188348.1)*	100
	BR68	*Indigofera frutescens*	*Burkholderia sp. MM6669-R2 (KM188348.1)*	100
	BR69	*Indigofera frutescens*	*Burkholderia sp. MM6669-R2 (KM188348.1)*	100
	CP588	*Indigofera frutescens*	*Burkholderia sp. MM5482-R2 (KF791743.1)*	100
	CP589	*Indigofera frutescens*	*Burkholderia sp. MM5482-R2 (KF791743.1)*	100
	CP590	*Indigofera frutescens*	*Burkholderia sp. MM5482-R2 (KF791743.1)*	100
	CP591	*Indigofera frutescens*	*Burkholderia sp. MM5482-R2 (KF791743.1)*	100
	BR534	*Indigofera mauritanica*	*Burkholderia sp. MM6669-R2 (KM188348.1)*	99
	BR535	*Indigofera mauritanica*	*Burkholderia sp. BL55-R2 (KM188420.1)*	100
	BR536	*Indigofera mauritanica*	*Burkholderia sp. BL55-R2 (KM188420.1)*	100
	BR537	*Indigofera mauritanica*	*Burkholderia sp. BL55-R2 (KM188420.1)*	100
	SM52	*Indigofera sarmentosa*	*Burkholderia sp. MM5496-R1 (KF791745.1)*	99
	BR103	*Indigofera sp.*	*Burkholderia sp. MM6669-R2 (KM188348.1)*	100
	BR152	*Indigofera sp.*	*Burkholderia sp. MM6669-R2 (KM188348.1)*	100
	PK221	*Lebeckia ambigua*	*Burkholderia sp. BL21-ind5-R2 (KM188387.1)*	100
	PK225	*Lebeckia ambigua*	*Burkholderia sp. BL21-ind5-R2 (KM188387.1)*	100
	RH602	*Lebeckia sp.*	*Burkholderia sp. MM5496-R1 (KF791745.1)*	100
	RH625	*Lebeckia sp.*	*Burkholderia sp. MM5496-R1 (KF791745.1)*	100
	RH614	*Podalyria argentea*	*Burkholderia sp. BL29-ind5-R2 (KM188409.1)*	99
	RH615	*Podalyria argentea*	*Burkholderia sp. BL29-ind5-R2 (KM188409.1)*	99
	RH616	*Podalyria argentea*	*Burkholderia sp. BL29-ind5-R2 (KM188409.1)*	99
	JH154	*Podalyria calyptrata*	*Burkholderia sp. BL18-ind8-R3 (KM188379.1)*	99
	JH160	*Podalyria calyptrata*	*Burkholderia sp. BL16-ind1-R2 (KM188366.1)*	99
	KM195	*Podalyria calyptrata*	*Burkholderia sp. BL29-ind6-R1 (KM188410.1)*	99
	KM198	*Podalyria calyptrata*	*Paraburkholderia sprentiae WSM5005 (CP017562.1)*	99

DNA region	Strain number	Legume host	Closest match (GenBank Accession No.)	% Similarity
	LH1	*Podalyria calyptrata*	*Burkholderia sp. MM6669-R2 (KM188348.1)*	100
	RM173	*Podalyria calyptrata*	*Burkholderia sp. BL18-ind8-R2 (KM188378.1)*	99
	RM175	*Podalyria calyptrata*	*Burkholderia sp. BL18-ind8-R2 (KM188378.1)*	100
	VG188	*Podalyria calyptrata*	*Burkholderia sp. BL21-ind4-R1 (KM188384.1)*	100
	VG193	*Podalyria calyptrata*	*Burkholderia sp. BL21-ind4-R1 (KM188384.1)*	100
	VG204	*Podalyria calyptrata*	*Burkholderia sp. BL16-ind1-R2 (KM188366.1)*	99
	VG206	*Podalyria calyptrata*	*Burkholderia sp. BL9-ind2-R2 (KM188351.1)*	100
	WB164	*Podalyria calyptrata*	*Burkholderia sp. BL21-ind4-R1 (KM188384.1)*	99
	WB168	*Podalyria calyptrata*	*Burkholderia sp. BL28-ind1-R3 (KM188428.1)*	99
	WB229	*Podalyria calyptrata*	*Burkholderia sp. BL29-ind6-R1 (KM188410.1)*	99
	WB232	*Podalyria calyptrata*	*Burkholderia sp. BL21-ind5-R2 (KM188387.1)*	100
	SE584	*Podalyria sericea*	*Burkholderia sp. BL13-R2 (KM188361.1)*	99
	LH7	*Podalyria sericea*	*Burkholderia sp. MM6669-R2 (KM188348.1)*	99
	SE585	*Podalyria sericea*	*Burkholderia sp. BL13-R2 (KM188361.1)*	99
	SE586	*Podalyria sericea*	*Burkholderia sp. BL13-R2 (KM188361.1)*	99
	SE587	*Podalyria sericea*	*Burkholderia sp. MM5482-R2 (KF791743.1)*	100
	LH22	*Virgilia oroboides*	*Burkholderia sp. MM5878 (KF840398.1)*	100
	LH23	*Virgilia oroboides*	*Burkholderia sp. MM5878 (KF840398.1)*	100
	SE538	*Virgilia oroboides*	*Burkholderia sp. BL17-R1 (KM188371.1)*	100
	SE539	*Virgilia oroboides*	*Burkholderia sp. BL17-R1 (KM188371.1)*	100
	SE540	*Virgilia oroboides*	*Burkholderia sp. BL17-R1 (KM188371.1)*	100
	SE541	*Virgilia oroboides*	*Burkholderia sp. BL17-R1 (KM188371.1)*	100
	TM140	*Virgilia oroboides*	*Burkholderia sp. BL17-R1 (KM188371.1)*	100
	RM600	*Argyrolobium lunare*	*Mesorhizobium sp. MM5369 (KF802693.1)*	98
	RM98	*Argyrolobium lunare*	*Mesorhizobium sp. OD13 (KF802674.1)*	99
	RM552	*Aspalathus cordata*	*Mesorhizobium sp. OD13 (KF802674.1)*	99
	SE561	*Aspalathus cordata*	*Mesorhizobium sp. OD13 (KF802674.1)*	99
	CP630	*Aspalathus ericifolia*	*Mesorhizobium sp. MM5734 (KF802683.1)*	99
	SE582	*Aspalathus ericifolia*	*Mesorhizobium sp. OD18 (KF802669.1)*	100
	BK312	*Psoralea fleta*	*Mesorhizobium sp. MM5343 (KF802700.1)*	99
	BK315	*Psoralea fleta*	*Mesorhizobium sp. MM5343 (KF802700.1)*	98
	RS178	*Psoralea pinnata*	*Mesorhizobium sp. MM5343 (KF802700.1)*	99
	BK308	*Psoralea usitata*	*Mesorhizobium sp. BL637 (KP013169.1)*	99

DNA region	Strain number	Legume host	Closest match (GenBank Accession No.)	% Similarity
recA	334N1	*Aspalathus carnosa*	*Burkholderia tuberum STM4252 (HE864382.1)*	99
	334N2	*Aspalathus carnosa*	*Burkholderia tuberum STM4252 (HE864382.1)*	99
	334N3	*Aspalathus carnosa*	*Burkholderia tuberum STM4252 (HE864382.1)*	99
	334N4	*Aspalathus carnosa*	*Burkholderia tuberum STM4252 (HE864382.1)*	99
	334N5	*Aspalathus carnosa*	*Burkholderia tuberum STM4252 (HE864382.1)*	99
	334N6	*Aspalathus carnosa*	*Burkholderia tuberum STM4252 (HE864382.1)*	99
	334N7	*Aspalathus carnosa*	*Burkholderia sp. WSM4175 (HE994066.1)*	99
	334N8	*Aspalathus carnosa*	*Burkholderia sp. MM6511-R2 (KF791816.1)*	99
	334N9	*Aspalathus carnosa*	*Burkholderia sp. MM6511-R2 (KF791816.1)*	99
	330N1	*Indigofera candolleana*	*Burkholderia sp. MM6511-R2 (KF791816.1)*	99
	330N2	*Indigofera candolleana*	*Burkholderia sp. MM5819 (KF791820.1)*	99
	330N3	*Indigofera candolleana*	*Burkholderia sp. MM5477-R2 (KF791799.1)*	99
	330N4	*Indigofera candolleana*	*Burkholderia sp. WSM4175 (HE994066.1)*	99
	330N5	*Indigofera candolleana*	*Burkholderia sp. WSM4175 (HE994066.1)*	99
	330N6	*Indigofera candolleana*	*Burkholderia sp. WSM4175 (HE994066.1)*	99
	330N7	*Indigofera candolleana*	*Burkholderia sp. WSM4175 (HE994066.1)*	99
	330N8	*Indigofera candolleana*	*Burkholderia sp. MM6511-R2 (KF791816.1)*	99
	330N9	*Indigofera candolleana*	*Burkholderia sp. MM6511-R2 (KF791816.1)*	99
	325N1	*Indigofera superba*	*Burkholderia sp. MM6511-R2 (KF791816.1)*	99
	325N2	*Indigofera superba*	*Burkholderia sp. MM6511-R2 (KF791816.1)*	99
	325N3	*Indigofera superba*	*Burkholderia sp. MM6511-R2 (KF791816.1)*	99
	325N4	*Indigofera superba*	*Burkholderia sp. MM6511-R2 (KF791816.1)*	99
	325N5	*Indigofera superba*	*Burkholderia sp. MM6511-R2 (KF791816.1)*	99
	325N6	*Indigofera superba*	*Burkholderia sp. MM6511-R2 (KF791816.1)*	99
	325N7	*Indigofera superba*	*Burkholderia sp. MM6511-R2 (KF791816.1)*	99

DNA region	Strain number	Legume host	Closest match (GenBank Accession No.)	% Similarity
	325NX	*Indigofera superba*	*Burkholderia sp. MM6463B (KF791848.1)*	99
	326N1	*Indigofera superba*	*Burkholderia sp. WSM4175 (HE994066.1)*	99
	326N1	*Indigofera superba*	*Burkholderia sp. WSM4175 (HE994066.1)*	99
	326N10	*Indigofera superba*	*Burkholderia sp. MM6463B (KF791848.1)*	99
	326N11	*Indigofera superba*	*Burkholderia sp. WSM4175 (HE994066.1)*	99
	326N12	*Indigofera superba*	*Burkholderia sp. WSM4175 (HE994066.1)*	99
	326N13	*Indigofera superba*	*Burkholderia sp. MM6463B (KF791848.1)*	99
	326N14	*Indigofera superba*	*Burkholderia sp. WSM4175 (HE994066.1)*	99
	326N15	*Indigofera superba*	*Burkholderia sp. WSM4175 (HE994066.1)*	99
	326N16	*Indigofera superba*	*Burkholderia sp. MM6511-R2 (KF791816.1)*	99
	326N17	*Indigofera superba*	*Burkholderia sp. OD25-R1 (KF791830.1)*	99
	326N18	*Indigofera superba*	*Burkholderia sp. WSM4175 (HE994066.1)*	99
	326N2	*Indigofera superba*	*Burkholderia sp. MM5477-R2 (KF791799.1)*	99
	326N2	*Indigofera superba*	*Burkholderia sp. MM6463B (KF791848.1)*	99
	326N3	*Indigofera superba*	*Burkholderia sp. MM6463B (KF791848.1)*	99
	326N3	*Indigofera superba*	*Burkholderia sp. WSM4175 (HE994066.1)*	99
	326N4	*Indigofera superba*	*Burkholderia sp. MM6463B (KF791848.1)*	99
	326N4	*Indigofera superba*	*Burkholderia sp. MM5477-R2 (KF791799.1)*	99
	326N5	*Indigofera superba*	*Burkholderia sp. WSM4175 (HE994066.1)*	99
	326N5	*Indigofera superba*	*Burkholderia sp. WSM4175 (HE994066.1)*	99
	326N6	*Indigofera superba*	*Burkholderia sp. WSM4175 (HE994066.1)*	99
	326N6	*Indigofera superba*	*Burkholderia sp. MM5477-R2 (KF791799.1)*	99
	326N7	*Indigofera superba*	*Burkholderia sp. MM6463B (KF791848.1)*	99
	326N7	*Indigofera superba*	*Burkholderia sp. OD25-R1 (KF791830.1)*	99
	326N8	*Indigofera superba*	*Burkholderia sp. WSM4175 (HE994066.1)*	99
	326N8	*Indigofera superba*	*Burkholderia sp. WSM4175 (HE994066.1)*	99

DNA region	Strain number	Legume host	Closest match (GenBank Accession No.)	% Similarity
	326N9	*Indigofera superba*	*Burkholderia sp. MM6463B (KF791848.1)*	99
	328N1	*Indigofera superba*	*Burkholderia sp. 973N7 (KT718935.1)*	99
	328N2	*Indigofera superba*	*Burkholderia sp. OD120 (KF791814.1)*	99
	328N3	*Indigofera superba*	*Burkholderia sp. 973N7 (KT718935.1)*	99
	328N4	*Indigofera superba*	*Burkholderia sp. 973N7 (KT718935.1)*	99
	328N5	*Indigofera superba*	*Burkholderia sp. MM5477-R2 (KF791799.1)*	99
	328N6	*Indigofera superba*	*Burkholderia sp. OD120 (KF791814.1)*	99
	328N7	*Indigofera superba*	*Burkholderia sp. 973N7 (KT718935.1)*	99
	328N8	*Indigofera superba*	*Burkholderia sp. OD120 (KF791814.1)*	98
	333N1	*Indigofera superba*	*Burkholderia sp. WSM4175 (HE994066.1)*	99
	333N10	*Indigofera superba*	*Burkholderia sp. 973N3 (KT718932.1)*	99
	333N11	*Indigofera superba*	*Burkholderia sp. WSM4175 (HE994066.1)*	99
	333N12	*Indigofera superba*	*Burkholderia sp. 973N7 (KT718935.1)*	99
	333N13	*Indigofera superba*	*Burkholderia sp. 973N7 (KT718935.1)*	99
	333N14	*Indigofera superba*	*Burkholderia sp. 973N3 (KT718932.1)*	99
	333N15	*Indigofera superba*	*Burkholderia sp. MM6463B (KF791848.1)*	99
	333N16	*Indigofera superba*	*Burkholderia sp. 973N7 (KT718935.1)*	99
	333N17	*Indigofera superba*	*Burkholderia sp. MM5477-R2 (KF791799.1)*	99
	333N18	*Indigofera superba*	*Burkholderia sp. WSM4175 (HE994066.1)*	99
	333N19	*Indigofera superba*	*Burkholderia sp. MM5819 (KF791820.1)*	99
	333N2	*Indigofera superba*	*Burkholderia sp. WSM4175 (HE994066.1)*	99
	333N20	*Indigofera superba*	*Burkholderia sp. 973N7 (KT718935.1)*	99
	333N21	*Indigofera superba*	*Burkholderia sp. MM5477-R2 (KF791799.1)*	99
	333N22	*Indigofera superba*	*Burkholderia sp. MM6463B (KF791848.1)*	99
	333N23	*Indigofera superba*	*Burkholderia sp. 973N7 (KT718935.1)*	99
	333N24	*Indigofera superba*	*Burkholderia sp. MM6463B (KF791848.1)*	99

DNA region	Strain number	Legume host	Closest match (GenBank Accession No.)	% Similarity
	333N25	*Indigofera superba*	*Burkholderia sp. 973N3 (KT718932.1)*	99
	333N26	*Indigofera superba*	*Burkholderia sp. 988N8 (KT718943.1)*	99
	333N27	*Indigofera superba*	*Burkholderia sp. MM5819 (KF791820.1)*	99
	333N3	*Indigofera superba*	*Burkholderia sp. WSM4175 (HE994066.1)*	99
	333N4	*Indigofera superba*	*Burkholderia sp. MM6463B (KF791848.1)*	99
	333N5	*Indigofera superba*	*Burkholderia sp. MM6463B (KF791848.1)*	99
	333N6	*Indigofera superba*	*Burkholderia sp. MM6463B (KF791848.1)*	99
	333N7	*Indigofera superba*	*Burkholderia sp. MM5477-R2 (KF791799.1)*	99
	333N8	*Indigofera superba*	*Burkholderia sp. 989N1 (KT718902.1)*	99
	333N9	*Indigofera superba*	*Burkholderia sp. 973N7 (KT718935.1)*	99
	327N10	*Psoralea pullata*	*Mesorhizobium sp. MM5343 (KF802791.1)*	99
	327N11	*Psoralea pullata*	*Mesorhizobium sp. MM5378 (KF802772.1)*	99
	327N3	*Psoralea pullata*	*Mesorhizobium sp. MM5413 (KF802790.1)*	99
	3237N4	*Psoralea pullata*	*Mesorhizobium sp. MM5343 (KF802791.1)*	99
	3237N5	*Psoralea pullata*	*Mesorhizobium sp. MM5413 (KF802790.1)*	99
	3237N6	*Psoralea pullata*	*Mesorhizobium sp. MM5413 (KF802790.1)*	99
	3237N7	*Psoralea pullata*	*Mesorhizobium sp. MM5343 (KF802791.1)*	100
	3237N8	*Psoralea pullata*	*Mesorhizobium sp. MM5343 (KF802791.1)*	100
nodC	334N1	*Aspalathus carnosa*	*Burkholderia sp. BL16 I4R2 (KR154710.1)*	99
	334N2	*Aspalathus carnosa*	*Burkholderia tuberum STM678 (AJ306730.1)*	99
	334N3	*Aspalathus carnosa*	*Burkholderia tuberum STM678 (AJ306730.1)*	99
	334N4	*Aspalathus carnosa*	*Burkholderia tuberum STM678 (AJ306730.1)*	99
	334N5	*Aspalathus carnosa*	*Burkholderia tuberum STM678 (AJ306730.1)*	99
	334N6	*Aspalathus carnosa*	*Burkholderia sp. BL16 I4R2 (KR154710.1)*	99
	334N7	*Aspalathus carnosa*	*Burkholderia tuberum STM678 (AJ306730.1)*	99
	334N8	*Aspalathus carnosa*	*Paraburkholderia sprentiae WSM5005 (CP017565.1)*	99
	334N9	*Aspalathus carnosa*	*Paraburkholderia sprentiae WSM5005 (CP017565.1)*	99
	325N3	*Indigofera superba*	*Paraburkholderia sprentiae WSM5005 (CP017565.1)*	99
	325N4	*Indigofera*	*Paraburkholderia sprentiae WSM5005*	99

DNA region	Strain number	Legume host	Closest match (GenBank Accession No.)	% Similarity
		superba	*(CP017565.1)*	
	325N5	*Indigofera superba*	*Paraburkholderia sprentiae WSM5005 (CP017565.1)*	99
	325N6	*Indigofera superba*	*Paraburkholderia sprentiae WSM5005 (CP017565.1)*	99
	325N7	*Indigofera superba*	*Paraburkholderia sprentiae WSM5005 (CP017565.1)*	99
	325NX	*Indigofera superba*	*Paraburkholderia sprentiae WSM5005 (CP017565.1)*	99
	326N1	*Indigofera superba*	*Burkholderia tuberum STM678 (AJ306730.1)*	99
	326N1	*Indigofera superba*	*Burkholderia tuberum STM678 (AJ306730.1)*	99
	326N10	*Indigofera superba*	*Burkholderia sp. BL23 I2R2 (KR154718.1)*	100
	326N11	*Indigofera superba*	*Burkholderia tuberum STM678 (AJ306730.1)*	99
	326N12	*Indigofera superba*	*Burkholderia tuberum STM678 (AJ306730.1)*	99
	326N13	*Indigofera superba*	*Burkholderia sp. OD123 (KP013138.1)*	98
	326N14	*Indigofera superba*	*Burkholderia tuberum STM678 (AJ306730.1)*	99
	326N15	*Indigofera superba*	*Burkholderia tuberum STM678 (AJ306730.1)*	99
	326N16	*Indigofera superba*	*Paraburkholderia sprentiae WSM5005 (CP017565.1)*	99
	326N17	*Indigofera superba*	*Paraburkholderia sprentiae WSM5005 (CP017565.1)*	99
	326N18	*Indigofera superba*	*Burkholderia tuberum STM678 (AJ306730.1)*	99
	326N2	*Indigofera superba*	*Burkholderia sp. OD123 (KP013138.1)*	99
	326N3	*Indigofera superba*	*Burkholderia sp. BL23 I2R2 (KR154718.1)*	99
	326N3	*Indigofera superba*	*Burkholderia tuberum STM678 (AJ306730.1)*	99
	326N4	*Indigofera superba*	*Burkholderia sp. BL13 R2 (KR154707.1)*	98
	326N5	*Indigofera superba*	*Burkholderia tuberum STM678 (AJ306730.1)*	99
	326N6	*Indigofera superba*	*Burkholderia tuberum STM678 (AJ306730.1)*	99
	326N7	*Indigofera superba*	*Paraburkholderia sprentiae WSM5005 (CP017565.1)*	99
	326N8	*Indigofera superba*	*Burkholderia tuberum STM678 (AJ306730.1)*	99
	326N8	*Indigofera superba*	*Paraburkholderia sprentiae WSM5005 (CP017565.1)*	99
	326N9	*Indigofera superba*	*Burkholderia sp. OD123 (KP013138.1)*	99

DNA region	Strain number	Legume host	Closest match (GenBank Accession No.)	% Similarity
	333N1	*Indigofera superba*	*Burkholderia tuberum STM678 (AJ306730.1)*	99
	333N10	*Indigofera superba*	*Burkholderia sp. 986N1 (KT718833.1)*	99
	333N11	*Indigofera superba*	*Burkholderia tuberum STM678 (AJ306730.1)*	99
	333N12	*Indigofera superba*	*Burkholderia sp. BL27 I3R6 (KR154722.1)*	99
	333N13	*Indigofera superba*	*Burkholderia sp. 986N1 (KT718833.1)*	99
	333N14	*Indigofera superba*	*Burkholderia sp. 986N1 (KT718833.1)*	99
	333N15	*Indigofera superba*	*Burkholderia sp. BL13 R2 (KR154707.1)*	99
	333N16	*Indigofera superba*	*Burkholderia sp. 973N7 (KT718871.1)*	99
	333N17	*Indigofera superba*	*Burkholderia tuberum STM678 (AJ306730.1)*	99
	333N18	*Indigofera superba*	*Burkholderia tuberum STM678 (AJ306730.1)*	99
	333N19	*Indigofera superba*	*Burkholderia tuberum STM678 (AJ306730.1)*	99
	333N2	*Indigofera superba*	*Burkholderia tuberum STM678 (AJ306730.1)*	99
	333N20	*Indigofera superba*	*Burkholderia sp. 986N1 (KT718833.1)*	99
	333N21	*Indigofera superba*	*Burkholderia tuberum STM678 (AJ306730.1)*	99
	333N22	*Indigofera superba*	*Burkholderia sp. BL23 I2R2 (KR154718.1)*	99
	333N23	*Indigofera superba*	*Burkholderia sp. 986N1 (KT718833.1)*	99
	333N24	*Indigofera superba*	*Burkholderia sp. BL23 I2R2 (KR154718.1)*	99
	333N25	*Indigofera superba*	*Burkholderia sp. 986N1 (KT718833.1)*	99
	333N26	*Indigofera superba*	*Burkholderia sp. BL16 I4R2 (KR154710.1)*	99
	333N27	*Indigofera superba*	*Burkholderia tuberum STM678 (AJ306730.1)*	99
	333N3	*Indigofera superba*	*Burkholderia tuberum STM678 (AJ306730.1)*	99
	333N4	*Indigofera superba*	*Burkholderia sp. BL23 I2R2 (KR154718.1)*	99
	333N5	*Indigofera superba*	*Burkholderia sp. BL23 I2R2 (KR154718.1)*	99
	333N7	*Indigofera superba*	*Burkholderia tuberum STM678 (AJ306730.1)*	99
	333N8	*Indigofera superba*	*Burkholderia sp. BL27 I3R6 (KR154722.1)*	99
	333N9	*Indigofera superba*	*Burkholderia sp. BL27 I3R6 (KR154722.1)*	99

DNA region	Strain number	Legume host	Closest match (GenBank Accession No.)	% Similarity
	327N1	*Psoralea pullata*	*Mesorhizobium sp. 998N23 (KT719013.1)*	98
	327N10	*Psoralea pullata*	*Mesorhizobium sp. 998N23 (KT719013.1)*	99
	327N11	*Psoralea pullata*	*Mesorhizobium sp. MM5357 (KR154632.1)*	99
	327N16	*Psoralea pullata*	*Mesorhizobium sp. 998N23 (KT719013.1)*	99
	327N17	*Psoralea pullata*	*Mesorhizobium sp. 998N23 (KT719013.1)*	99
	327N18	*Psoralea pullata*	*Mesorhizobium sp. 998N23 (KT719013.1)*	99
	327N19	*Psoralea pullata*	*Mesorhizobium sp. 998N23 (KT719013.1)*	99
	327N2	*Psoralea pullata*	*Mesorhizobium sp. 998N23 (KT719013.1)*	99
	327N2	*Psoralea pullata*	*Mesorhizobium sp. 998N23 (KT719013.1)*	99
	327N20	*Psoralea pullata*	*Mesorhizobium sp. MM5462 (KR154635.1)*	99
	327N21	*Psoralea pullata*	*Mesorhizobium sp. 998N23 (KT719013.1)*	99
	327N22	*Psoralea pullata*	*Mesorhizobium sp. 998N23 (KT719013.1)*	99
	327N23	*Psoralea pullata*	*Mesorhizobium sp. 998N23 (KT719013.1)*	99
	327N24	*Psoralea pullata*	*Mesorhizobium sp. 998N23 (KT719013.1)*	98
	327N25	*Psoralea pullata*	*Mesorhizobium sp. 998N23 (KT719013.1)*	98
	327N26	*Psoralea pullata*	*Mesorhizobium sp. ICMP 12638 (KM018083.1)*	100
	327N3	*Psoralea pullata*	*Mesorhizobium sp. MM5357 (KR154632.1)*	99
	327N3	*Psoralea pullata*	*Mesorhizobium sp. 998N23 (KT719013.1)*	99
	327N4	*Psoralea pullata*	*Mesorhizobium sp. MM5462 (KR154635.1)*	98
	327N4	*Psoralea pullata*	*Mesorhizobium sp. 998N23 (KT719013.1)*	98
	327N5	*Psoralea pullata*	*Mesorhizobium sp. MM5357 (KR154632.1)*	99
	327N6	*Psoralea pullata*		
	327N7	*Psoralea pullata*		
	327N8	*Psoralea pullata*		
	329bN1	*Psoralea restioides*		
	329bN2	*Psoralea restioides*		
	329bN3	*Psoralea restioides*		
	329bN4	*Psoralea restioides*		
	329bN5	*Psoralea restioides*		
	329bN6	*Psoralea restioides*		

Table S2. List of rhizobial strains with binary scoring of their soil types. A score of 0 indicates that a strain was not found in that particular soil type, while a score of 1 means that it was.

Genus	Strain	Granite	Limestone	Sandstone	Shale
Burkholderia	*Burkholderia sp. (Amphithalea ericifolia MM5482)*	0	0	1	0
	Burkholderia sp. (Aspalathus argyrella SE571)	0	0	1	0
	Burkholderia sp. (Aspalathus argyrella SE572)	0	0	1	0
	Burkholderia sp. (Aspalathus callosa MM5477)	0	0	1	0
	Burkholderia sp. (Aspalathus capensis CPT36)	0	0	1	0
	Burkholderia sp. (Aspalathus carnosa MM5496)	0	0	1	0
	Burkholderia sp. (Aspalathus carnosa SW5)	0	0	1	0
	Burkholderia sp. (Boolusafra bituminosa OD29)	0	0	1	0
	Burkholderia sp. (Crotalaria sp. OD120)	0	0	1	0
	Burkholderia sp. (Dipogon lignosus LH6)	1	0	0	0
	Burkholderia sp. (Indigofera angustifolia MM5878)	0	0	1	0
	Burkholderia sp. (Indigofera candolleana LH4)	1	0	0	0
	Burkholderia sp. (Indigofera cytisoides MM5819)	0	0	1	0
	Burkholderia sp. (Indigofera filifolia SE555)	0	0	1	0
	Burkholderia sp. (Indigofera filifolia SE556)	0	0	1	0
	Burkholderia sp. (Indigofera filifolia SE557)	0	0	1	0
	Burkholderia sp. (Indigofera filifolia VG182)	0	0	1	0
	Burkholderia sp. (Indigofera filifolia VG185)	0	0	1	0
	Burkholderia sp. (Indigofera filiformis BR62)	1	0	0	0
	Burkholderia sp. (Indigofera frutescens BR542)	1	0	0	0
	Burkholderia sp. (Indigofera frutescens BR543)	1	0	0	0
	Burkholderia sp. (Indigofera frutescens BR544)	1	0	0	0
	Burkholderia sp. (Indigofera frutescens BR545)	1	0	0	0
	Burkholderia sp. (Indigofera frutescens BR67)	1	0	0	0
	Burkholderia sp. (Indigofera frutescens BR68)	1	0	0	0
	Burkholderia sp. (Indigofera frutescens BR69)	1	0	0	0
	Burkholderia sp. (Indigofera frutescens CP 589)	0	0	1	0
	Burkholderia sp. (Indigofera frutescens CP588)	0	0	1	0
	Burkholderia sp. (Indigofera frutescens CP590)	0	0	1	0
	Burkholderia sp. (Indigofera frutescens CP591)	0	0	1	0
	Burkholderia sp. (Indigofera mauritanica BR534)	1	0	0	0
	Burkholderia sp. (Indigofera mauritanica BR535)	1	0	0	0
	Burkholderia sp. (Indigofera mauritanica BR536)	1	0	0	0
	Burkholderia sp. (Indigofera mauritanica BR537)	1	0	0	0
	Burkholderia sp. (Indigofera sarmentosa SE573)	0	0	1	0
	Burkholderia sp. (Indigofera sarmentosa SM52)	0	0	1	0
	Burkholderia sp. (Indigofera sp. BR103)	1	0	0	0
	Burkholderia sp. (Indigofera sp. BR152)	1	0	0	0
	Burkholderia sp. (Indigofera sp. MM5746)	0	0	1	0
	Burkholderia sp. (Lebeckia ambigua PK221)	0	0	1	0
	Burkholderia sp. (Lebeckia ambigua PK225)	0	0	1	0
	Burkholderia sp. (Lebeckia sp. RH601)	0	0	1	0
	Burkholderia sp. (Lebeckia sp. RH602)	0	0	1	0

Genus	Strain	Granite	Limestone	Sandstone	Shale
	Burkholderia sp. (Lebeckia sp. RH625)	0	0	1	0
	Burkholderia sp. (Liparia splendens SE574)	0	0	1	0
	Burkholderia sp. (Podalyria argentea RH614)	0	0	1	0
	Burkholderia sp. (Podalyria argentea RH615)	0	0	1	0
	Burkholderia sp. (Podalyria argentea RH616)	0	0	1	0
	Burkholderia sp. (Podalyria burchellii MM5875)	0	0	1	0
	Burkholderia sp. (Podalyria calyptrata JH154)	0	0	1	0
	Burkholderia sp. (Podalyria calyptrata JH160)	0	0	1	0
	Burkholderia sp. (Podalyria calyptrata KM195)	0	0	1	0
	Burkholderia sp. (Podalyria calyptrata KM198)	0	0	1	0
	Burkholderia sp. (Podalyria calyptrata LH1)	1	0	0	0
	Burkholderia sp. (Podalyria calyptrata MM5337)	0	0	1	0
	Burkholderia sp. (Podalyria calyptrata OD25)	0	0	1	0
	Burkholderia sp. (Podalyria calyptrata RM1730	0	0	0	1
	Burkholderia sp. (Podalyria calyptrata RM175)	0	0	0	1
	Burkholderia sp. (Podalyria calyptrata VG188)	0	0	1	0
	Burkholderia sp. (Podalyria calyptrata VG193)	0	0	1	0
	Burkholderia sp. (Podalyria calyptrata VG204)	0	0	1	0
	Burkholderia sp. (Podalyria calyptrata VG205)	0	0	1	0
	Burkholderia sp. (Podalyria calyptrata VG206)	0	0	1	0
	Burkholderia sp. (Podalyria calyptrata WB164)	0	0	1	0
	Burkholderia sp. (Podalyria calyptrata WB168)	0	0	1	0
	Burkholderia sp. (Podalyria calyptrata WB229)	0	0	1	0
	Burkholderia sp. (Podalyria calyptrata WB232)	0	0	1	0
	Burkholderia sp. (Podalyria sericea LH7)	1	0	0	0
	Burkholderia sp. (Podalyria sericea MM5384)	1	0	0	0
	Burkholderia sp. (Podalyria sericea SE584)	0	0	1	0
	Burkholderia sp. (Podalyria sericea SE585)	0	0	1	0
	Burkholderia sp. (Podalyria sericea SE586)	0	0	1	0
	Burkholderia sp. (Podalyria sericea SE587)	0	0	1	0
	Burkholderia sp. (Rafnia acuminata OD22)	0	0	1	0
	Burkholderia sp. (Rafnia sp. OD28)	0	0	1	0
	Burkholderia sp. (Virgilia oroboides LH22)	1	0	0	0
	Burkholderia sp. (Virgilia oroboides LH23)	1	0	0	0
	Burkholderia sp. (Virgilia oroboides MM5366)	0	0	1	0
	Burkholderia sp. (Virgilia oroboides SE538)	0	0	1	0
	Burkholderia sp. (Virgilia oroboides SE539)	0	0	1	0
	Burkholderia sp. (Virgilia oroboides SE540)	0	0	1	0
	Burkholderia sp. (Virgilia oroboides SE541)	0	0	1	0
	Burkholderia sp. (Virgilia oroboides TM140)	0	0	1	0
Mesorhizobium	*Mesorhizobium sp. (Argyrolobium lunare MM5369)*	0	0	1	0
	Mesorhizobium sp. (Argyrolobium lunare OD14)	0	0	1	0
	Mesorhizobium sp. (Argyrolobium lunare OD48)	0	1	0	0
	Mesorhizobium sp. (Argyrolobium lunare RM5810)	0	0	0	1

Genus	Strain	Granite	Limestone	Sandstone	Shale
	Mesorhizobium sp. (Argyrolobium lunare RM600)	0	0	0	1
	Mesorhizobium sp. (Argyrolobium lunare RM98)	0	0	0	1
	Mesorhizobium sp. (Argyrolobium velutinum OD47)	0	1	0	0
	Mesorhizobium sp. (Aspalathuis astroites OD18)	0	0	1	0
	Mesorhizobium sp. (Aspalathus aurantiaca MM5397)	0	0	1	0
	Mesorhizobium sp. (Aspalathus bracteata MM5618)	1	0	0	0
	Mesorhizobium sp. (Aspalathus chenopoda BR621)	1	0	0	0
	Mesorhizobium sp. (Aspalathus chenopoda BR623)	1	0	0	0
	Mesorhizobium sp. (Aspalathus chenopoda BR624)	1	0	0	0
	Mesorhizobium sp. (Aspalathus chenopoda RM559)	0	0	0	1
	Mesorhizobium sp. (Aspalathus ciliaris CS13166)	0	0	1	0
	Mesorhizobium sp. (Aspalathus ciliaris MM5361)	0	0	1	0
	Mesorhizobium sp. (Aspalathus ciliaris OD108)	0	0	1	0
	Mesorhizobium sp. (Aspalathus cordata OD13)	0	0	1	0
	Mesorhizobium sp. (Aspalathus cordata RM552)	0	0	0	1
	Mesorhizobium sp. (Aspalathus cordata SE551)	0	0	1	0
	Mesorhizobium sp. (Aspalathus cordata SE561)	0	0	1	0
	Mesorhizobium sp. (Aspalathus cordata SE563)	0	0	1	0
	Mesorhizobium sp. (Aspalathus ericifolia BR604)	1	0	0	0
	Mesorhizobium sp. (Aspalathus ericifolia BR605)	1	0	0	0
	Mesorhizobium sp. (Aspalathus ericifolia CP627)	0	0	1	0
	Mesorhizobium sp. (Aspalathus ericifolia CP628)	0	0	1	0
	Mesorhizobium sp. (Aspalathus ericifolia CP630)	0	0	1	0
	Mesorhizobium sp. (Aspalathus ericifolia MM5352)	0	0	1	0
	Mesorhizobium sp. (Aspalathus ericifolia OD31)	0	1	0	0
	Mesorhizobium sp. (Aspalathus ericifolia SE582)	0	0	1	0
	Mesorhizobium sp. (Aspalathus ericifolia SE598)	0	0	1	0
	Mesorhizobium sp. (Aspalathus ericifolia SE599)	0	0	1	0
	Mesorhizobium sp. (Aspalathus hispida RM564)	0	0	0	1
	Mesorhizobium sp. (Aspalathus spicata MM5398)	0	0	1	0
	Mesorhizobium sp. (Aspalathus spicata MM5440)	0	0	1	0
	Mesorhizobium sp. (Aspalathus uniflora MM5734)	0	0	1	0
	Mesorhizobium sp. (Aspalathus uniflora OD26)	0	0	1	0
	Mesorhizobium sp. (Indigofera angustifolia MM5378)	1	0	0	0
	Mesorhizobium sp. (Indigofera venusta MM5377)	0	0	1	0
	Mesorhizobium sp. (Otholobium bracteolatum OD42)	0	1	0	0
	Mesorhizobium sp. (Otholobium fruticans BR619)	1	0	0	0
	Mesorhizobium sp. (Otholobium fruticans BR620)	1	0	0	0
	Mesorhizobium sp. (Otholobium hirtum LH609)	1	0	0	0
	Mesorhizobium sp. (Otholobium hirtum LH610)	1	0	0	0
	Mesorhizobium sp. (Otholobium hirtum LH611)	1	0	0	0

Genus	Strain	Granite	Limestone	Sandstone	Shale
	Mesorhizobium sp. (Otholobium hirtum LH612)	1	0	0	0
	Mesorhizobium sp. (Otholobium hirtum MM5334)	0	0	0	1
	Mesorhizobium sp. (Otholobium hirtum MM5376)	1	0	0	0
	Mesorhizobium sp. (Otholobium hirtum MM5382)	0	0	1	0
	Mesorhizobium sp. (Otholobium hirtum OD32)	0	1	0	0
	Mesorhizobium sp. (Otholobium hirtum RM579)	0	0	0	1
	Mesorhizobium sp. (OtHOLOBIUM obliquum MM5370)	0	0	1	0
	Mesorhizobium sp. (Otholobium virgatum MM5333)	0	0	0	1
	Mesorhizobium sp. (Otholobium virgatum MM5357)	0	0	1	0
	Mesorhizobium sp. (Otholobium virgatum RM567)	0	0	0	1
	Mesorhizobium sp. (Otholobium virgatum SW44)	0	0	1	0
	Mesorhizobium sp. (Otholobium zeyheri MM5675)	0	0	1	0
	Mesorhizobium sp. (Psoralea aphylla SE595)	0	0	1	0
	Mesorhizobium sp. (Psoralea aphylla SE596)	0	0	1	0
	Mesorhizobium sp. (Psoralea aphylla SE597)	0	0	1	0
	Mesorhizobium sp. (Psoralea asarina MM5360)	0	0	1	0
	Mesorhizobium sp. (Psoralea asarina OD15)	0	0	1	0
	Mesorhizobium sp. (Psoralea asarina RM548)	0	0	0	1
	Mesorhizobium sp. (Psoralea brilliantissima OD52)	0	1	0	0
	Mesorhizobium sp. (Psoralea congesta MM5462)	0	0	1	0
	Mesorhizobium sp. (Psoralea fleta BK312)	0	0	1	0
	Mesorhizobium sp. (Psoralea fleta BK315)	0	0	1	0
	Mesorhizobium sp. (Psoralea laxa OD119)	0	0	0	1
	Mesorhizobium sp. (Psoralea oligophylla OD118)	0	0	0	1
	Mesorhizobium sp. (Psoralea pinnata RH607)	0	0	1	0
	Mesorhizobium sp. (Psoralea pinnata RH608)	0	0	1	0
	Mesorhizobium sp. (Psoralea pinnata RM569)	0	0	0	1
	Mesorhizobium sp. (Psoralea pinnata RS176)	0	0	1	0
	Mesorhizobium sp. (Psoralea pinnata RS178)	0	0	1	0
	Mesorhizobium sp. (Psoralea pinnata SE592)	0	0	1	0
	Mesorhizobium sp. (Psoralea rigidula MM5343)	0	0	1	0
	Mesorhizobium sp. (Psoralea usitata BK308)	0	0	1	0

Table S3. List of rhizobial strains used with the altitude and pH data of their sites.

Genus	Strain	Altitude	pH
Burkholderia	*Burkholderia sp. (Amphithalea ericifolia MM5482)*	20	4.64
	Burkholderia sp. (Aspalathus argyrella SE571)	486	4.97
	Burkholderia sp. (Aspalathus argyrella SE572)	487	4.97
	Burkholderia sp. (Aspalathus callosa MM5477)	10	4.62
	Burkholderia sp. (Aspalathus capensis CPT36)	254	4.07
	Burkholderia sp. (Aspalathus carnosa MM5496)	57	3.73
	Burkholderia sp. (Aspalathus carnosa SW5)	112	3.53
	Burkholderia sp. (Boolusafra bituminosa OD29)	272	3.18
	Burkholderia sp. (Crotalaria sp. OD120)	240	4.64
	Burkholderia sp. (Dipogon lignosus LH6)	228	5.1
	Burkholderia sp. (Indigofera angustifolia MM5878)	40	4.5
	Burkholderia sp. (Indigofera candolleana LH4)	228	5.1
	Burkholderia sp. (Indigofera cytisoides MM5819)	179	4.36
	Burkholderia sp. (Indigofera filifolia SE555)	296	4.97
	Burkholderia sp. (Indigofera filifolia SE556)	296	4.97
	Burkholderia sp. (Indigofera filifolia SE557)	296	4.97
	Burkholderia sp. (Indigofera filifolia VG182)	240	3.43
	Burkholderia sp. (Indigofera filifolia VG185)	240	3.43
	Burkholderia sp. (Indigofera filiformis BR62)	410	4.4
	Burkholderia sp. (Indigofera frutescens BR542)	412	4.4
	Burkholderia sp. (Indigofera frutescens BR543)	412	4.4
	Burkholderia sp. (Indigofera frutescens BR544)	412	4.4
	Burkholderia sp. (Indigofera frutescens BR545)	412	4.4
	Burkholderia sp. (Indigofera frutescens BR67)	404	4.4
	Burkholderia sp. (Indigofera frutescens BR68)	408	4.4
	Burkholderia sp. (Indigofera frutescens BR69)	408	4.4
	Burkholderia sp. (Indigofera frutescens CP589)	413	4.4
	Burkholderia sp. (Indigofera frutescens CP588)	413	4.4
	Burkholderia sp. (Indigofera frutescens CP590)	413	4.4
	Burkholderia sp. (Indigofera frutescens CP591)	413	4.1
	Burkholderia sp. (Indigofera mauritanica BR534)	414	4.4
	Burkholderia sp. (Indigofera mauritanica BR535)	414	4.4
	Burkholderia sp. (Indigofera mauritanica BR536)	414	4.4
	Burkholderia sp. (Indigofera mauritanica BR537)	414	4.4
	Burkholderia sp. (Indigofera sarmentosa SE573)	296	4.97
	Burkholderia sp. (Indigofera sarmentosa SM52)	112	3.53
	Burkholderia sp. (Indigofera sp. BR103)	411	4.4
	Burkholderia sp. (Indigofera sp. BR152)	416	4.46
	Burkholderia sp. (Indigofera sp. MM5746)	296	4.18
	Burkholderia sp. (Lebeckia ambigua PK221)	447	3.56
	Burkholderia sp. (Lebeckia ambigua PK225)	447	3.56
	Burkholderia sp. (Lebeckia sp. RH601)	369	3.83
	Burkholderia sp. (Lebeckia sp. RH602)	369	3.83
	Burkholderia sp. (Lebeckia sp. RH625)	365	3.83

Genus	Strain	Altitude	pH
	Burkholderia sp. (Liparia splendens SE574)	296	4.97
	Burkholderia sp. (Podalyria argentea RH614)	364	3.83
	Burkholderia sp. (Podalyria argentea RH615)	364	3.83
	Burkholderia sp. (Podalyria argentea RH616)	364	3.83
	Burkholderia sp. (Podalyria burchellii MM5875)	28	4.5
	Burkholderia sp. (Podalyria calyptrata JH154)	296	4.18
	Burkholderia sp. (Podalyria calyptrata JH160)	296	4.18
	Burkholderia sp. (Podalyria calyptrata KM195)	128	3.76
	Burkholderia sp. (Podalyria calyptrata KM198)	128	3.76
	Burkholderia sp. (Podalyria calyptrata LH1)	228	5.1
	Burkholderia sp. (Podalyria calyptrata MM5337)	420	4.56
	Burkholderia sp. (Podalyria calyptrata OD25)	272	3.62
	Burkholderia sp. (Podalyria calyptrata RM173)	166	4.4
	Burkholderia sp. (Podalyria calyptrata RM175)	166	4.4
	Burkholderia sp. (Podalyria calyptrata VG188)	241	3.43
	Burkholderia sp. (Podalyria calyptrata VG193)	241	3.43
	Burkholderia sp. (Podalyria calyptrata VG204)	241	3.43
	Burkholderia sp. (Podalyria calyptrata VG205)	241	3.43
	Burkholderia sp. (Podalyria calyptrata VG206)	241	3.43
	Burkholderia sp. (Podalyria calyptrata WB164)	84	3.56
	Burkholderia sp. (Podalyria calyptrata WB168)	84	3.56
	Burkholderia sp. (Podalyria calyptrata WB229)	87	3.59
	Burkholderia sp. (Podalyria calyptrata WB232)	87	3.59
	Burkholderia sp. (Podalyria sericea LH7)	228	5.1
	Burkholderia sp. (Podalyria sericea MM5384)	91	5.26
	Burkholderia sp. (Podalyria sericea SE584)	312	4.97
	Burkholderia sp. (Podalyria sericea SE585)	312	4.97
	Burkholderia sp. (Podalyria sericea SE586)	312	4.97
	Burkholderia sp. (Podalyria sericea SE587)	312	4.97
	Burkholderia sp. (Rafnia acuminata OD22)	272	3.62
	Burkholderia sp. (Rafnia sp. OD28)	272	3.62
	Burkholderia sp. (Virgilia oroboides LH22)	174	5.1
	Burkholderia sp. (Virgilia oroboides LH23)	174	5.1
	Burkholderia sp. (Virgilia oroboides MM5366)	296	4.18
	Burkholderia sp. (Virgilia oroboides SE538)	313	4.97
	Burkholderia sp. (Virgilia oroboides SE539)	313	4.97
	Burkholderia sp. (Virgilia oroboides SE540)	313	4.97
	Burkholderia sp. (Virgilia oroboides SE541)	313	4.97
	Burkholderia sp. (Virgilia oroboides TM140)	686	4.97
Mesorhizobium	*Mesorhizobium sp. (Argyrolobium lunare MM5369)*	296	4.18
	Mesorhizobium sp. (Argyrolobium lunare OD14)	296	3.18
	Mesorhizobium sp. (Argyrolobium lunare OD48)	11.4	6.39
	Mesorhizobium sp. (Argyrolobium lunare RM581)	212	4.4
	Mesorhizobium sp. (Argyrolobium lunare RM600)	219	4.4
	Mesorhizobium sp. (Argyrolobium lunare RM98)	218	4.4

Genus	Strain	Altitude	pH
	Mesorhizobium sp. (Argyrolobium velutinum OD47)	11.4	6.71
	Mesorhizobium sp. (Aspalathuis astroites OD18)	296	4
	Mesorhizobium sp. (Aspalathus aurantiaca MM5397)	368	4.11
	Mesorhizobium sp. (Aspalathus bracteata MM5618)	582	5.19
	Mesorhizobium sp. (Aspalathus chenopoda BR621)	415	4.4
	Mesorhizobium sp. (Aspalathus chenopoda BR623)	415	4.4
	Mesorhizobium sp. (Aspalathus chenopoda BR624)	415	4.4
	Mesorhizobium sp. (Aspalathus chenopoda RM559)	215	4.4
	Mesorhizobium sp. (Aspalathus ciliaris CS13166)	40	4.1
	Mesorhizobium sp. (Aspalathus ciliaris MM5361)	296	4
	Mesorhizobium sp. (Aspalathus ciliaris OD108)	522	4.76
	Mesorhizobium sp. (Aspalathus cordata OD13)	296	4
	Mesorhizobium sp. (Aspalathus cordata RM552)	217	4.4
	Mesorhizobium sp. (Aspalathus cordata SE551)	296	4.97
	Mesorhizobium sp. (Aspalathus cordata SE561)	298	4.97
	Mesorhizobium sp. (Aspalathus cordata SE563)	292	4.97
	Mesorhizobium sp. (Aspalathus ericifolia BR604)	415	4.4
	Mesorhizobium sp. (Aspalathus ericifolia BR605)	141	4.4
	Mesorhizobium sp. (Aspalathus ericifolia CP627)	114	4.07
	Mesorhizobium sp. (Aspalathus ericifolia CP628)	114	4.07
	Mesorhizobium sp. (Aspalathus ericifolia CP630)	114	4.07
	Mesorhizobium sp. (Aspalathus ericifolia MM5352)	272	4.88
	Mesorhizobium sp. (Aspalathus ericifolia OD31)	581	5.19
	Mesorhizobium sp. (Aspalathus ericifolia SE582)	312	4.97
	Mesorhizobium sp. (Aspalathus ericifolia SE598)	311	4.97
	Mesorhizobium sp. (Aspalathus ericifolia SE599)	311	4.97
	Mesorhizobium sp. (Aspalathus hispida RM564)	205	5.47
	Mesorhizobium sp. (Aspalathus spicata MM5398)	368	4.11
	Mesorhizobium sp. (Aspalathus spicata MM5440)	229	3.9
	Mesorhizobium sp. (Aspalathus uniflora MM5734)	348	3.49
	Mesorhizobium sp. (Aspalathus uniflora OD26)	272	3.62
	Mesorhizobium sp. (Indigofera angustifolia MM5378)	68	4.5
	Mesorhizobium sp. (Indigofera venusta MM5377)	68	4.5
	Mesorhizobium sp. (Otholobium bracteolatum OD42)	11.4	6.71
	Mesorhizobium sp. (Otholobium fruticans BR619)	636	4.4
	Mesorhizobium sp. (Otholobium fruticans BR620)	363	4.4
	Mesorhizobium sp. (Otholobium hirtum LH609)	472	4.77
	Mesorhizobium sp. (Otholobium hirtum LH610)	472	4.77
	Mesorhizobium sp. (Otholobium hirtum LH611)	472	4.77
	Mesorhizobium sp. (Otholobium hirtum LH612)	472	4.77
	Mesorhizobium sp. (Otholobium hirtum MM5334)	203	5.31
	Mesorhizobium sp. (Otholobium hirtum MM5376)	68	4.9
	Mesorhizobium sp. (Otholobium hirtum MM5382)	68	4.59
	Mesorhizobium sp. (Otholobium hirtum OD32)	581	5.19
	Mesorhizobium sp. (Otholobium hirtum RM579)	247	4.4
	Mesorhizobium sp. (OtHOLOBIUM obliquum MM5370)	330	4.18

Genus	Strain	Altitude	pH
	Mesorhizobium sp. (Otholobium virgatum MM5333)	203	5.31
	Mesorhizobium sp. (Otholobium virgatum MM5357)	296	4.26
	Mesorhizobium sp. (Otholobium virgatum RM567)	247	4.4
	Mesorhizobium sp. (Otholobium virgatum SW44)	124	3.86
	Mesorhizobium sp. (Otholobium zeyheri MM5675)	610	5.39
	Mesorhizobium sp. (Psoralea aphylla SE595)	317	4.97
	Mesorhizobium sp. (Psoralea aphylla SE596)	317	4.97
	Mesorhizobium sp. (Psoralea aphylla SE597)	317	4.97
	Mesorhizobium sp. (Psoralea asarina MM5360)	296	4.26
	Mesorhizobium sp. (Psoralea asarina OD15)	296	3.18
	Mesorhizobium sp. (Psoralea asarina RM548)	162	4.4
	Mesorhizobium sp. (Psoralea brilliantissima OD52)	107	6.5
	Mesorhizobium sp. (Psoralea congesta MM5462)	20	4.5
	Mesorhizobium sp. (Psoralea fleta BK312)	358	3.29
	Mesorhizobium sp. (Psoralea fleta BK315)	358	3.29
	Mesorhizobium sp. (Psoralea laxa OD119)	240	4.64
	Mesorhizobium sp. (Psoralea oligophylla OD118)	240	4.64
	Mesorhizobium sp. (Psoralea pinnata RH607)	364	3.83
	Mesorhizobium sp. (Psoralea pinnata RH608)	364	3.83
	Mesorhizobium sp. (Psoralea pinnata RM569)	210	4.4
	Mesorhizobium sp. (Psoralea pinnata RS176)	136	3.84
	Mesorhizobium sp. (Psoralea pinnata RS178)	136	3.84
	Mesorhizobium sp. (Psoralea pinnata SE592)	321	4.97
	Mesorhizobium sp. (Psoralea rigidula MM5343)	753	3.29
	Mesorhizobium sp. (Psoralea usitata BK308)	356	3.29
